# Supplementary figures and images for: All-you-can-eat buffet: A spider-specialized bat species (Myotis emarginatus) turns into a pest fly eater around cattle
Source: PLoS One. 2024 May 8;19(5):e0302028. doi: 10.1371/journal.pone.0302028 (PMC11078406; doi:10.1371/journal.pone.0302028)

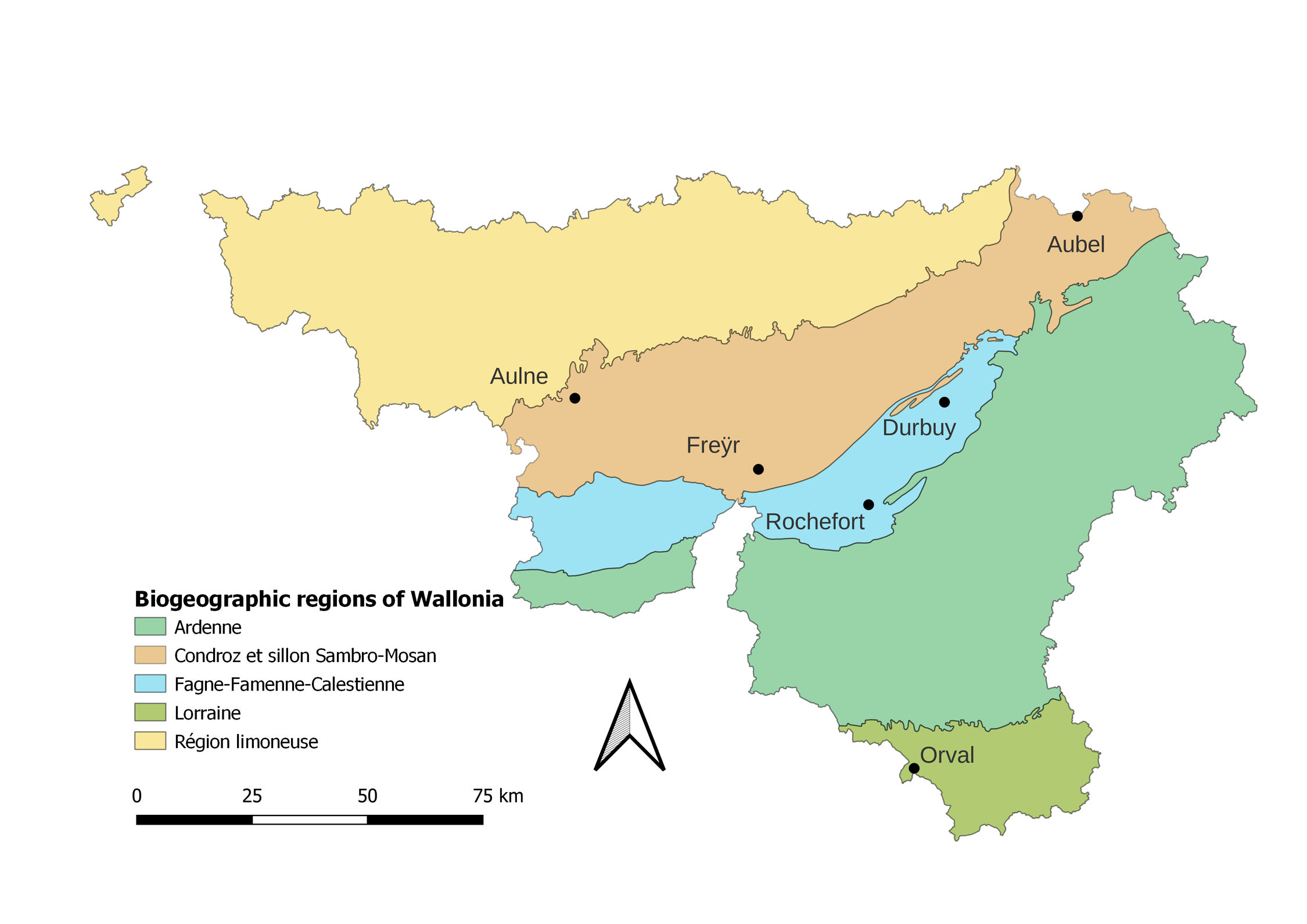

Supplement: S1 Fig — Location of the six sampled maternity colonies of Myotis emarginatus studied for their diet in Wallonia, Belgium. (TIF) [file pone.0302028.s001.tif]

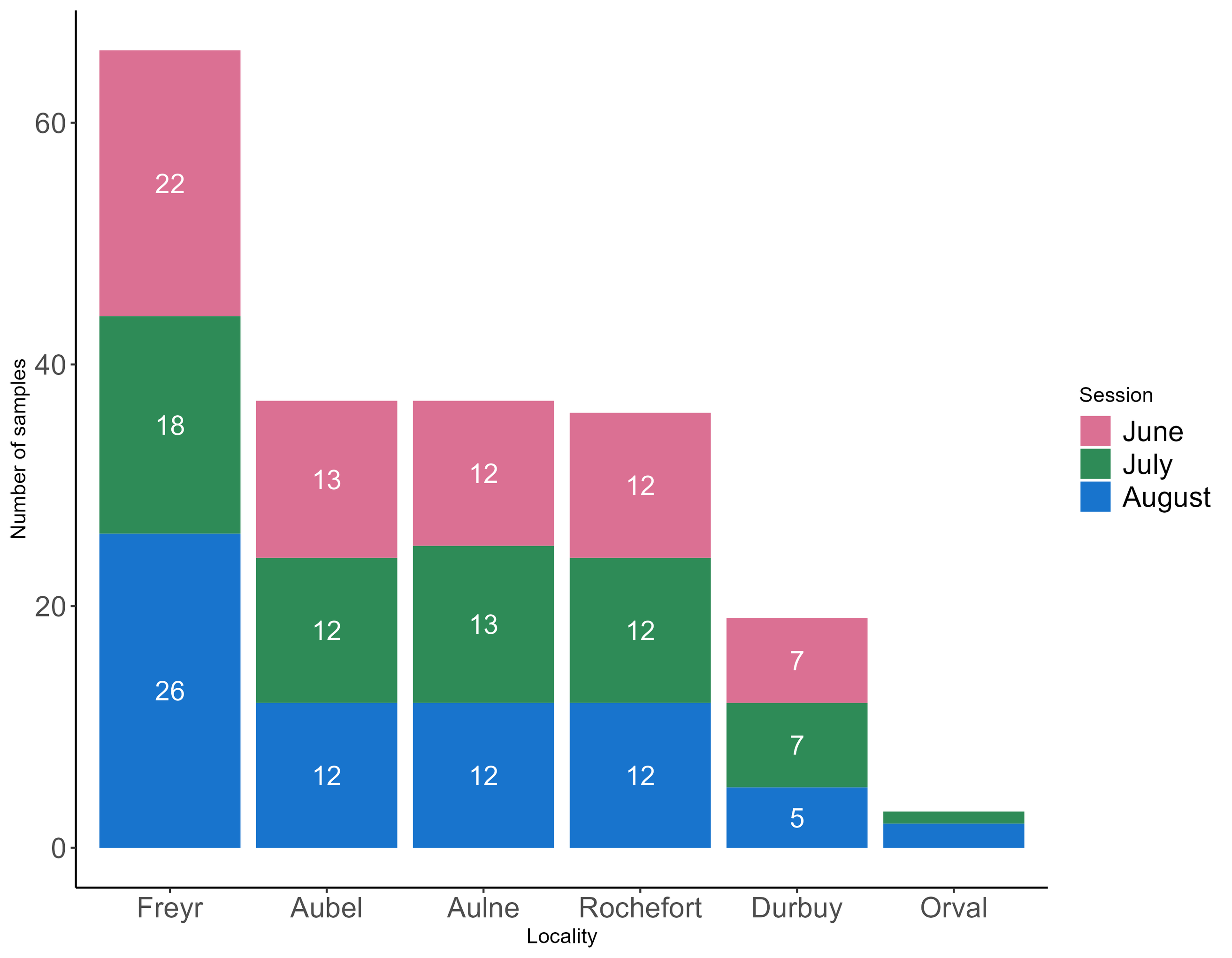

Supplement: S2 Fig — (TIF) [file pone.0302028.s002.tif]

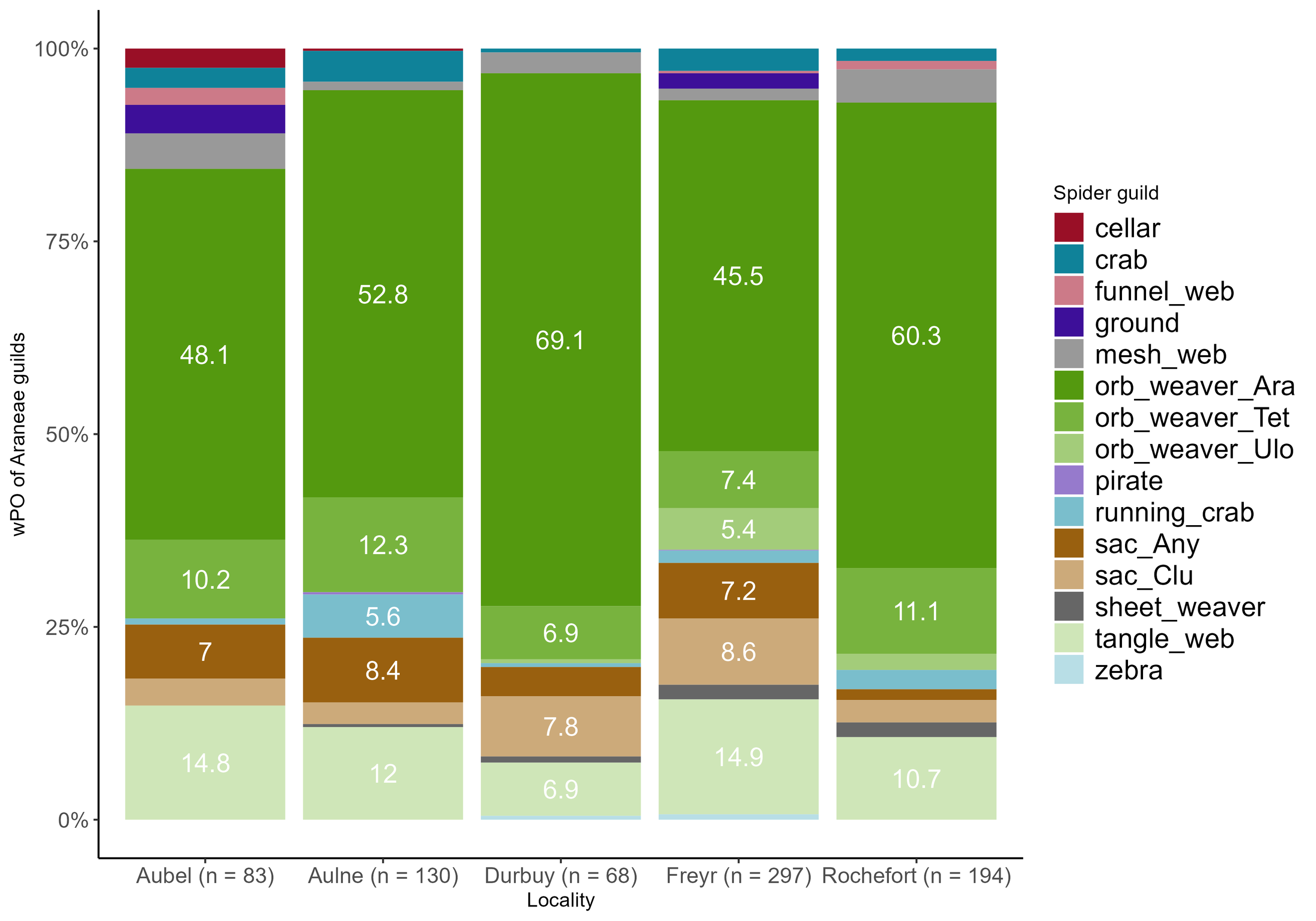

Supplement: S3 Fig — weighted percentage of occurrence (wPO: Within each sample, it is the prey item occurrence/total number of occurrences of all prey*100) of the Araneae taxa according to their spider guilds (based on the classification developed by Uetz et al. in 1999 [89]), in the diet of each sampled colony of Myotis emarginatus. wPO values above 5% were displayed on the plot. In the orb-weaver spiders, Ara, Tet and Ulo stands for the families Araneidae, Tetragnathidae and Uloboridae, respectively. Abbreviations of the spider guilds are detailed in the S1 File. On the x-axis, n = number of taxa occurrences. (TIF) [file pone.0302028.s003.tif]

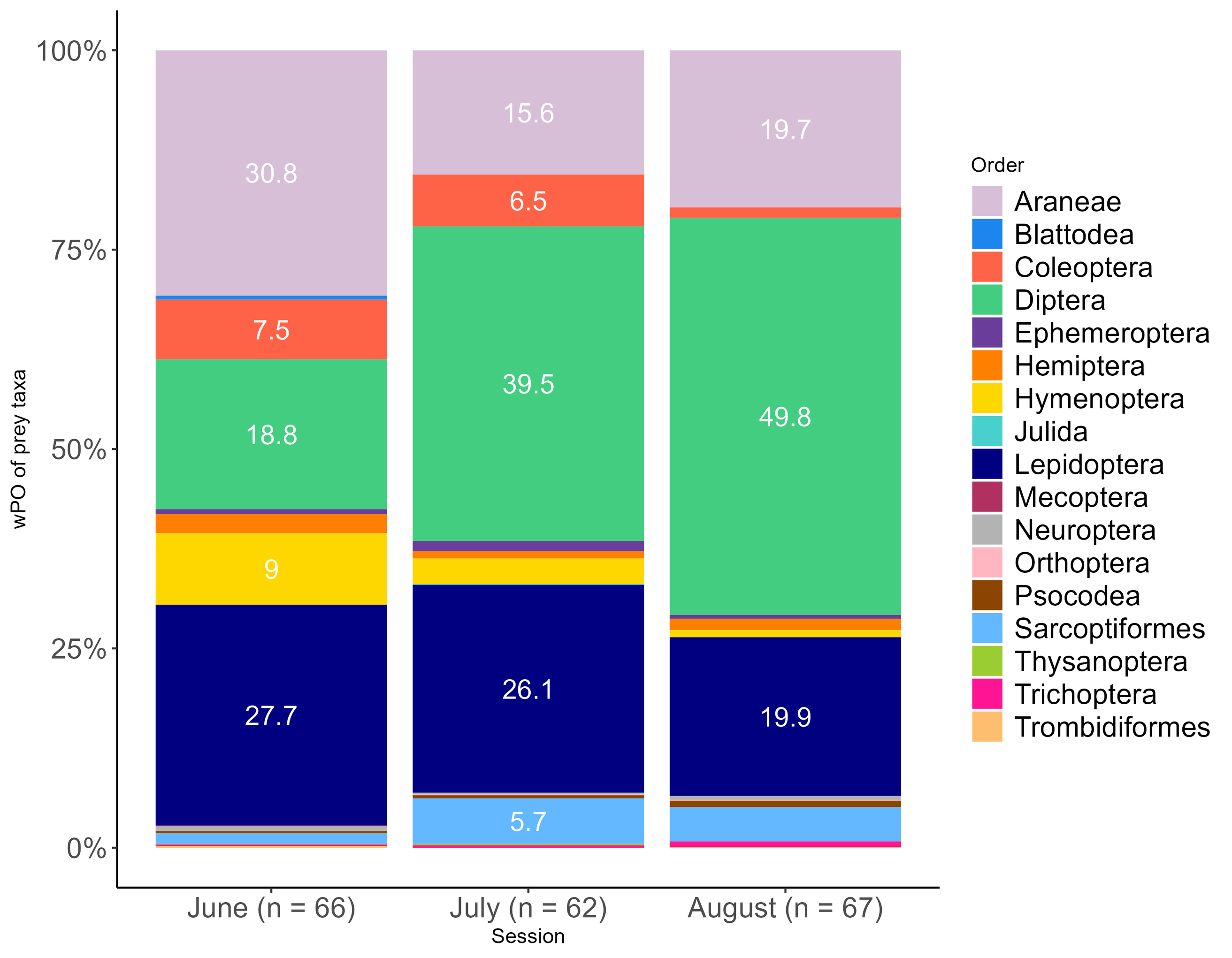

Supplement: S4 Fig — weighted percentage of occurrence (wPO: Within each sample, it is the prey item occurrence/total number of occurrences of all prey*100) of the prey taxa eaten by Myotis emarginatus according to their taxonomic order and to the sampling session (June, July, S3). wPO values above 5% were displayed on the plot. On the x-axis, n = number of individual fecal pellets. (TIF) [file pone.0302028.s004.tif]
