# Supplementary material for: All-you-can-eat buffet: A spider-specialized bat species (Myotis emarginatus) turns into a pest fly eater around cattle
Source: PLoS One. 2024 May 8;19(5):e0302028. doi: 10.1371/journal.pone.0302028 (PMC11078406; doi:10.1371/journal.pone.0302028)
Supplement: S1 File — (PDF) [file pone.0302028.s012.pdf]

S1 File: Detailed material and methods

A. Study sites and guano collection

Breeding colonies

The six initial sites have been selected to cover the broadest possible range for this species in Belgium, which delimits the northern edge of its range. All nursery roosts were located in buildings being at least 25 km apart, thereby ensuring no exchange of individuals occurred between them, as inferred from maximum distances flown by females during the breeding season [1–3]. The exact count of bats was determined by taking a photograph of the bat colony. During the first sampling session (June), the colony from Durbuy hosted less than 50 *Myotis emarginatus* (hereafter abbreviated as "ME") individual bats, the ones from Aubel and Freyr between 100 and 200 ME and the ones from Aulne and Rochefort between 200 and 300 ME. In Orval, ME colony size was unknown since bats were hidden in a chimney. While the maternity colonies from Aubel, Aulne and Rochefort were monospecific, those from Freyr, Durbuy and Orval hosted both ME and *Rhinolophus ferrumequinum*, which is a common bat species association [4,5].

Study area

To analyze the landscape composition, we used the land cover classes defined by Radoux et al. [6] under the LifeWatch project. Those classes were combined into nine which are relevant for ME ecology. Below is explained how the initial classes were combined/renamed.

1) *Water bodies* was renamed as "Water".
2) *Natural Material Surfaces with less than 10% vegetation* was renamed as "Natural Material Surfaces <10% vegetation".
3) *Artificially sealed ground surface* & *Building, specific structures and facilities* were combined into "Artificially sealed grounds & buildings".
4) *Herbaceous in rotation during the year (e.g. crops)* was renamed as "Crops".
5) Grassland with intensive management was used without modification.
6) *Grassland and scrub of biological interest* & *Inundated grassland and scrub of biological interest* were combined into "Grasslands and scrubs of biological interest".
7) *Vegetation of recently disturbed area (e.g. clear cut)* was renamed as "Vegetation of recently disturbed areas".
8) *Coniferous trees (>= 3 m)* & *Small coniferous trees (< 3 m)* were combined into "Coniferous trees".
9) *Broadleaved trees (>= 3 m)* & *Small broadleaved trees (< 3 m) and shrubs* were combined into "Broadleaved trees".

The study area straddled two biogeographical regions [7]: Atlantic (Aulne) and Continental (all others), where a temperate weather occurred. Mean annual precipitations ranged from 850 to 960 mm [8]. All breeding colonies were at overall low altitude, ranging from 100 to 250 m above sea level. Water streams of various sizes – from a creek near Aubel to the Meuse River bordering the Freyr colony – were present near (< 1 km) each roost.

Sample collection

We avoided excessive stress on bats by installing the sampling plastic boxes in the evening—following bat emergence whenever possible—and by quickly removing them the next morning for sample collection outside of the roost.

B. DNA extraction, PCR amplification and sequencing

Extraction

DNA was extracted using a QIAamp® Fast DNA Stool Mini Kit (Qiagen, Hilden, Germany; Handbook version 02/2020), according to the manufacturer's protocol for Isolation of DNA from Stool for Pathogen Detection with some modifications to improve yield of Gram-positive bacteria, as the same DNA material was used in another study focusing on bat microbiota. Following step two (Inhibitex® addition), bead-beating was performed in a TissueLyser II device (Qiagen) at 30 Hz for 3 min. During step three, thermic lysis was increased to 95°C. Steps five to 14 were automated in a QIAcube® system (Qiagen). DNA was eluted in 90 µL ATE buffer.

PCR amplification

We conducted a two-step PCR strategy. A first step (PCR_1) aimed at amplifying the extracted DNA. The second step (PCR_2) was carried out to assign a unique combination of multiplexing tags to each PCR_1 product in order to pool (multiplex) them while being able to reassign each sequence to its sample of origin afterwards (demultiplexing).

As primers, we chose both the 133 bp fragment adapted by Galan et al. [9] from the primer pair developed by Gillet et al. [10] (MG-LCO1490-MiSeq and modified MG-univR-MiSeq as recommended in Galan et al.'s discussion) and the 157 bp minibarcode described by Zeale et al. [11] (ZBJ-ArtF1c and ZBJ-ArtR2c). These primers are hereafter referred to as "Galan" and "Zeale".

We choose Zeale primers [11] because they have been widely used to retrieve European insectivorous bat diets and are therefore associated with well-developed reference databases, thereby allowing the comparison with previous studies. However, these primers are highly biased toward Diptera and Lepidoptera [12] because their level of degeneracy is low. To counteract this bias, and because Tournayre and her colleagues [12] have shown that short and highly degenerated primers are critical to uncover ME diet, we have selected as a second primer set the one developed by Galan et al. [13], which targets a wide range of arthropods consumed by bats [12,14]. On top of that, this primer pair also amplifies bat DNA, making it extremely interesting to discriminate guano originating from our target species from the *R. ferrumequinum*'s feces in the mixed colonies. The PCR_1 primers were made of three sections: a partial overhang Illumina sequencing primers in the 5'-end, followed by a heterogeneity spacer and the target-specific primer. The heterogeneity spacer consisted of 0 to 6 bp of DNA inserted to minimize the issues associated with low sequence diversity in Illumina amplicon sequencing, as these several versions of each primer created an artificial nucleotide diversity, thereby enhancing the discrimination of clusters on the flow cell and the subsequent quality of reads.

In PCR_2, we incorporated a sample-specific combination of 8 bp long Illumina tags (i5 and i7) and Illumina sequencing adapters (P5 and P7) at the 5'-ends of PCR_1 amplicons, using the Nextera XT index kit (Illumina, San Diego, CA).

Samples preparation and sequencing

Following each PCR step, PCR products were bead-purified using Agencourt AMPure XP beads (Beckman Coulter Life Sciences, IN, USA) at a 0.8:1 amplified-DNA:bead ratio. Purified products from PCR_2 were subsequently fluorometrically quantified using a Quant-iT™ PicoGreen® dsDNA Assay Kit (Thermo Scientific, MA, USA) on a fluorimeter (FilterMax F3, Molecular Devices). Quantified products were then pooled in equimolarity per marker (5 ng/µL for Galan and 3 ng/µL for Zeale). Finally, for each primer pair, a total of 292 samples,

36 field blanks, 31 extraction blanks and 7 PCR_1 blanks were sequenced on an Illumina NovaSeq flow cell using 150 bp paired-end chemistry, a ≥ 5% PhiX control spike-in and targeting 100,000 reads per sample.

C. Bioinformatics and prey list construction

Denoising, merging and chimera removal with dada2: parameters

Regarding dada2 options in QIIME 2 [15], we choose the 'pseudo' pooling method, in which denoising is performed in two steps for an enhanced sensitivity to rare variants. We selected the 'pooled' chimera method whereby detection of chimeras is preceded by pooling of all reads. The maximum number of expected errors per read was set to 0.609 for the forward reads and to 1 for the reverse reads. Reads shorter than 100/106 bp (forward/reverse) were discarded.

Database construction, classifier training and taxonomic assignment

A custom BOLD database was curated using the method described by O'Rourke with some modifications [16] (see their project's GitHub repository: [17]). Briefly, arthropod records were collected from the BOLD (Barcode Of Life Data System; [18]) database through the bold R package [19] on 30 September 2022. Then, we applied a custom script so that only arthropod data matching the marker code "COI-5P" and for which taxonomic information was at least described to the family level was retained. Contrary to the method developed by O'Rourke, dereplication of the custom database was carried out with vsearch program exclusively [20], and not through the pick_otus.py script implemented in the QIIME 1 environment as it has become outdated.

Dereplicated database was finally imported into the QIIME 2 environment to be trained against both Galan's and Zeale's sequences (outputs of dada2) with the fit-classifier-naive-bayes method from the feature-classifier plugin [21,22], generating two distinct scikit-learn Naive Bayes classifiers. Then, the taxonomy of the ASVs was assigned using the trained classifiers with the classify-sklearn method within the same plugin (kmer-based machine learning; [21,22]).

Data filtering

Following the taxonomy assignment, we used the taxa plugin and the filter-seqs method to retain sequences with sufficient taxonomic information (i.e. sequences at least determined at the order level) and the filter-features method from the feature-table plugin to retain ASVs whose assignment confidence was ≥ 0.98. Through this filtering process, for the Galan and Zeale primers, we discarded 26,318 and 188 ASVs, respectively, as they corresponded to Chordata taxa (for Galan only) and due to extremely sparse taxonomic information (i.e. sequences at the kingdom-, phylum- or class- level) and 733 and 589 ASVs were further removed because of low confidence of assignment (< 0.98). To further deal with read redundancy, ASVs assigned to the same taxa and at the same taxonomic level were pooled.

After trimming the sequences present in blanks in R, we discarded biological items that did not occur in the study area and within a 500 km radius around it, arthropod taxa that represented potential contamination and those that, while being part of the diet, were not the actual targeted prey, such as aquatic taxa that were probably ingested when drinking water (Daphniidae family within the Branchiopoda class) and the consumed beetles' parasites (e.g. some members of the Mesostigmata and Sarcoptiformes orders within the Arachnida class). Since the Galan primers detect Chordata DNA, it allowed us to discard the samples originating from *R. ferrumequinum* to exclusively work on ME samples. The maximum contamination level reached 0.09% of read count in a ME sample from Freyr (166 reads of *R.*

ferrumequinum and 186,214 reads of ME). As this level was very low, no samples were discarded due to species contamination.

In R, we applied three additional filtering steps based on read abundance to minimize potential false-positives, contaminations and the detection of secondary predation. We first discarded samples for which the total read count was < 10^{-5} of the whole dataset read count to avoid large dissimilarity in sequencing depth across samples. We next removed—within each sample—all taxa occurrences for which the read count was < 10^{-4} of the total read count of that sample. Finally, we discarded taxa for which the overall read count was < 10 across all samples.

D. Statistical analyses

1. Datasets used for statistical analyses

It is worth mentioning that FTO (frequency of taxa occurrence) only quantifies the detection of prey taxa (PA data) and not actual scores of feeding events, as no reliable estimation of item abundance can be made from molecular reads data [23]. FSO (frequency of sample occurrence) has been shown to indicate the prey relative abundance across the landscape [24]. wPO (weighted percentage of occurrence) offers the advantage to generate clearer signals than read counts when it comes to diversified diet [25].

For data manipulations and computations, we used the tibble [26], purr [27], hablar [28], raster [29], dplyr [30], stringr [31], gdata [32], scales [33] and forcats [34] packages. For graphical representations, we made use of the following packages: graphics, ggplot2 [35] , car [36], gridExtra [37], pals [38] and ggpubr [39].

2. Variations throughout the breeding season

Regarding the Hill numbers calculations, they were based on incidence data (PA data). We drew accumulation curves based on sample richness, which corresponds to q = 0 for Hill numbers. We considered the diversity curves to be significantly different if the 95% confidence intervals, built with 500 bootstraps, did not overlap.

As concerns the GLMMs, we fitted a model to assess the session impact for each taxon order counting at least 20 prey taxa occurrences across all samples. We determined significant differences between models with the Anova function of the car package [36].

In regard to the PERMANOVA analyses, we applied the adonis2 function from the vegan package [40]. Pairwise differences (pairwise PERMANOVAs) between sessions were determined using the pairwise.adonis2 function in the pairwiseAdonis package [41]. For both adonis2 and pairwise.adonis2, statistical significance was assessed using 10,000 random permutations.

In running the NMDS, we set the trymax parameter—the maximum number of initial random attempts to find a stable solution—to 30. The most stable solution obtained a stress value of 0.242.

3. Ecological traits of arthropod prey species

The spider guilds classification [42] discriminates spiders according to their hunting strategy and web structure. In this study, we recovered 15 families belonging to 12 spider guilds, which are described in the following table.

Abbreviation	Spider guild	Family	Abbreviation	Spider guild	Family
orb_weaver_Ara	Orb-weaver spiders	Araneidae	funnel_web	Funnel-web spiders	Agelenidae
orb_weaver_Tet	Orb-weaver spiders	Tetragnathidae	tangle_web	Tangle_web spiders	Theridiidae
orb_weaver_Ulo	Orb-weaver spiders	Uloboridae	sheet_weaver	Sheet-weaver spiders	Linyphiidae
sac_Any	Sac spiders	Anyphaenidae	running_crab	Running crab spiders	Philodromidae
sac_Clu	Sac spiders	Clubionidae	crab	Crab spiders	Thomisidae
mesh_web	Mesh-web spiders	Dictynidae	zebra	Zebra spiders	Salticidae
cellar	Cellar spiders	Pholcidae	pirate	Pirate spiders	Mimetidae
ground	Ground runner spiders	Gnaphosidae			

4. Pest species consumption

Minor pests are those whose damages are either occasional or regular but which, in the latter case, are not entailed with economic losses. Major pests are those that regularly impact their host, thereby causing damages entailed with potentially high economic losses. Pathogen vectors/Livestock pests are the pests that may be harmful to plants or warm-blooded animals, either by their simple presence (infestations) or because they vehiculate parasites or pathogens.

References

1. Zahn A, Bauer S, Kriner E, Holzhaider J. Foraging habitats of *Myotis emarginatus* in Central Europe. Eur J Wildl Res. 2010;56: 395–400. doi:10.1007/s10344-009-0331-y

2. Goiti U, Aihartza J, Guiu M, Salsamendi E, Almenar D, Napal M, et al. Geoffroy's bat, *Myotis emarginatus*, preys preferentially on spiders in multistratified dense habitats: a study of foraging bats in the Mediterranean. Folia Zool. 2011;60: 17–24. doi:10.25225/fozo.v60.i1.a3.2011

3. Dekker JJ, Regelink JR, Jansen EA, Brinkmann R, Limpens H. Habitat use by female Geoffroy's bats (*Myotis emarginatus*) at its two northernmost maternity roosts and the implications for their conservation. Lutra. 2013;56: 111–120.

4. Flaquer C, Puig-Montserrat X, Burgas A, Russo D. Habitat selection by Geoffroy's bats (*Myotis emarginatus*) in a rural Mediterranean landscape: implications for conservation. Acta Chiropt. 2008;10: 61–67. doi:10.3161/150811008X331090

5. Dietz M, Pir JB, Hillen J. Does the survival of greater horseshoe bats and Geoffroy's bats in Western Europe depend on traditional cultural landscapes? Biodivers Conserv. 2013;22: 3007–3025. doi:10.1007/s10531-013-0567-4

6. Radoux J, Bourdouxhe A, Coppée T, De Vroey M, Dufrêne M, Defourny P. A Consistent Land Cover Map Time Series at 2 m Spatial Resolution—The LifeWatch 2006-2015-2018-2019 Dataset for Wallonia. Data. 2023;8: 13. doi:10.3390/data8010013

7. Biogeographical regions in Europe. 2016. Available: https://www.eea.europa.eu/data-and-maps/figures/biogeographical-regions-in-europe-2

8. Climat dans votre commune. In: L'Institut royal météorologique [Internet]. [cited 17 Jan 2024]. Available: https://www.meteo.be/fr/climat/climat-de-la-belgique/climat-dans-votre-commune

9. Galan M, Pons J-B, Tournayre O, Pierre É, Leuchtmann M, Pontier D, et al. Metabarcoding for the parallel identification of several hundred predators and their prey: Application to bat species diet analysis. Mol Ecol Resour. 2018;18: 474–489. doi:10.1111/1755-0998.12749

10. Gillet F, Tiouchichine M-L, Galan M, Blan F, Némoz M, Aulagnier S, et al. A new method to identify the endangered Pyrenean desman (*Galemys pyrenaicus*) and to study its diet, using next generation sequencing from faeces. Mamm Biol. 2015;80: 505–509. doi:10.1016/j.mambio.2015.08.002

11. Zeale MRK, Butlin RK, Barker GLA, Lees DC, Jones G. Taxon-specific PCR for DNA barcoding arthropod prey in bat faeces. Mol Ecol Resour. 2011;11: 236–244. doi:10.1111/j.1755-0998.2010.02920.x

12. Tournayre O, Leuchtmann M, Filippi-Codaccioni O, Trillat M, Piry S, Pontier D, et al. In silico and empirical evaluation of twelve metabarcoding primer sets for insectivorous diet analyses. Ecol Evol. 2020;10: 6310–6332. doi:10.1002/ece3.6362

13. Galan M, Razzauti M, Bard E, Bernard M, Brouat C, Charbonnel N, et al. 16S rRNA Amplicon Sequencing for Epidemiological Surveys of Bacteria in Wildlife. Bik H, editor. mSystems. 2016;1: e00032-16, /msys/1/4/e00032-16.atom. doi:10.1128/mSystems.00032-16

14. Corse E, Tougard C, Archambaud-Suard G, Agnèse J-F, Mandeng FDM, Bilong CFB, et al. One-locus-several-primers: A strategy to improve the taxonomic and haplotypic coverage in diet metabarcoding studies. Ecol Evol. 2019;9: 4603–4620. doi:10.1002/ece3.5063

15. Callahan BJ, McMurdie PJ, Rosen MJ, Han AW, Johnson AJA, Holmes SP. DADA2: High-resolution sample inference from Illumina amplicon data. Nat Methods. 2016;13: 581–583. doi:10.1038/nmeth.3869

16. O'Rourke DR, Bokulich NA, Jusino MA, MacManes MD, Foster JT. A total crapshoot? Evaluating bioinformatic decisions in animal diet metabarcoding analyses. Ecol Evol. 2020;10: 9721–9739. doi:10.1002/ece3.6594

17. O'Rourke DR. tidybug/docs/database_construction.md. In: GitHub [Internet]. 2020 [cited 14 Sep 2023]. Available: https://github.com/devonorourke/tidybug/blob/master/docs/sequence_filtering.md

18. Ratnasingham S, Hebert PDN. bold: The Barcode of Life Data System (http://www.barcodinglife.org). Molecular Ecology Notes. 2007;7: 355–364. doi:10.1111/j.1471-8286.2007.01678.x

19. Dubois S, Chamberlain S. bold: Interface to Bold Systems API. 2023. Available: https://cran.r-project.org/web/packages/bold/index.html

20. Rognes T, Flouri T, Nichols B, Quince C, Mahé F. VSEARCH: a versatile open source tool for metagenomic. Peer Journal. 2016. p. e2584. doi:10.7717/peerj.2584

21. Bokulich NA, Kaehler BD, Rideout JR, Dillon M, Bolyen E, Knight R, et al. Optimizing taxonomic classification of marker-gene amplicon sequences with QIIME 2's q2-feature-classifier plugin. Microbiome. 2018;6: 90. doi:10.1186/s40168-018-0470-z

22. Pedregosa F, Varoquaux G, Gramfort A, Michel V, Thirion B, Grisel O, et al. Scikit-learn: Machine learning in Python. J Mach Learn Res. 2011;12: 2825–2830.

23. Deagle BE, Thomas AC, McInnes JC, Clarke LJ, Vesterinen EJ, Clare EL, et al. Counting with DNA in metabarcoding studies: How should we convert sequence reads to dietary data? Mol Ecol. 2019;28: 391–406. doi:10.1111/mec.14734

24. Maslo B, Mau RL, Kerwin K, McDonough R, McHale E, Foster JT. Bats provide a critical ecosystem service by consuming a large diversity of agricultural pest insects. Agric Ecosyst Environ. 2022;324: 107722. doi:10.1016/j.agee.2021.107722

25. Andriollo T, Gillet F, Michaux JR, Ruedi M. The menu varies with metabarcoding practices: A case study with the bat *Plecotus auritus*. Gorokhova E, editor. PLoS ONE. 2019;14: e0219135. doi:10.1371/journal.pone.0219135

26. Müller K, Wickham H, Francois R, Bryan J, RStudio. tibble: Simple Data Frames. 2023. Available: https://cran.r-project.org/web/packages/tibble/index.html

27. Wickham H, Henry L, RStudio. purrr: Functional Programming Tools. 2023. Available: https://cran.r-project.org/web/packages/purrr/index.html

28. Sjoberg D. hablar: Non-Astonishing Results in R. 2023. Available: https://cran.r-project.org/web/packages/hablar/index.html

29. Hijmans RJ, Etten J van, Sumner M, Cheng J, Baston D, Bevan A, et al. raster: Geographic Data Analysis and Modeling. 2023. Available: https://cran.r-project.org/web/packages/raster/index.html

30. Wickham H, François R, Henry L, Müller K, Vaughan D, Software P, et al. dplyr: A Grammar of Data Manipulation. 2023. Available: https://cran.r-project.org/web/packages/dplyr/index.html

31. Wickham H, RStudio. stringr: Simple, Consistent Wrappers for Common String Operations. 2022. Available: https://cran.r-project.org/web/packages/stringr/index.html

32. Warnes GR, Gorjanc G, Magnusson A, Andronic L, Rogers J, MacQueen D, et al. gdata: Various R Programming Tools for Data Manipulation. 2023. Available: https://cran.r-project.org/web/packages/gdata/index.html

33. Wickham H, Seidel D, RStudio. scales: Scale Functions for Visualization. 2022. Available: https://cran.r-project.org/web/packages/scales/index.html

34. Wickham H, RStudio. forcats: Tools for Working with Categorical Variables (Factors). 2023. Available: https://cran.r-project.org/web/packages/forcats/index.html

35. Wickham H, Chang W, Henry L, Pedersen TL, Takahashi K, Wilke C, et al. ggplot2: Create Elegant Data Visualisations Using the Grammar of Graphics. 2023. Available: https://cran.r-project.org/web/packages/ggplot2/index.html

36. Fox J, Weisberg S, Price B, Adler D, Bates D, Baud-Bovy G, et al. car: Companion to Applied Regression. 2019. Available: https://cran.r-project.org/web/packages/car/index.html

37. Auguie B, Antonov A. gridExtra: Miscellaneous Functions for "Grid" Graphics. 2017. Available: https://cran.r-project.org/web/packages/gridExtra/index.html

38. Wright K. pals: Color Palettes, Colormaps, and Tools to Evaluate Them. 2021. Available: https://cran.r-project.org/web/packages/pals/index.html

39. Kassambara A. ggpubr: "ggplot2" Based Publication Ready Plots. 2023. Available: https://cran.r-project.org/web/packages/ggpubr/index.html

40. Oksanen J, Blanchet FG, Kindt R, Legendre P, Minchin PR, O'hara R, et al. Package 'vegan.' Community ecology package, version. 2022;2: 1–295.

41. Martinez Arbizu P. pairwiseAdonis. 2020. Available: https://github.com/pmartinezarbizu/pairwiseAdonis

42. Uetz GW, Halaj J, Cady AB. Guild structure of spiders in major crops. J Arachnol. 1999; 270–280.
