# Supplementary material for: All-you-can-eat buffet: A spider-specialized bat species (Myotis emarginatus) turns into a pest fly eater around cattle
Source: PLoS One. 2024 May 8;19(5):e0302028. doi: 10.1371/journal.pone.0302028 (PMC11078406; doi:10.1371/journal.pone.0302028)
Supplement: S2 File — (PDF) [file pone.0302028.s013.pdf]

S2 File: List of references used to determine the Araneae hunting strategy and web structure [1 to 3] and the Lepidoptera habitat use and life cycle [4 to 9].

[1–9]

1. Observations.be. In: Observations.be [Internet]. 2023 [cited 15 Jun 2023]. Available: https://observations.be/

2. Uetz GW, Halaj J, Cady AB. Guild structure of spiders in major crops. J Arachnol. 1999; 270–280.

3. Roberts M. Spiders of Britain and Northern Europe. Field guide. London: Harper Collins; 2001.

4. Waring P, Townsend M. Field Guide to the Moths of Great Britain and Ireland. 3rd ed. London: Bloomsbury Publishing Plc; 2017.

5. Sterling P, Parsons M. Field Guide to the Micro-Moths of Great Britain and Ireland. Bloomsbury USA; 2018.

6. Flemish Entomological Society. Catalogue of the Lepidoptera of Belgium. 2023 [cited 14 Jun 2023]. Available: https://projects.biodiversity.be/lepidoptera/

7. Bestimmung von Schmetterlingen und ihren Präimaginalstadien. In: Lepiforum e.V. [Internet]. 2023 [cited 14 Jun 2023]. Available: https://lepiforum.org/

8. Lurettigh J-M, Vandromme D, Demergès D. Macrohétérocères (Papillons de nuit). In: Oreina, Les papillons de France [Internet]. 2023 [cited 14 Jun 2023]. Available: https://oreina.org/artemisiae/observatoire/index.php?d=hetero

9. Muus TST, Corver SC. Soortenlijst van alle in Nederland voorkomende micro's. In: Microlepidoptera.nl, atlas van de kleine vlinders in Nederland. [Internet]. 2023 [cited 14 Jun 2023]. Available: http://www.microvlinders.nl/soorten/soortenlijst.php?fbclid=IwAR05jGW_LdU2bBLiLgcF5FhHJY8czZIMNoIfyIu67hyKaieoh-QOfbV93SY
