# Supplementary material for: All-you-can-eat buffet: A spider-specialized bat species (Myotis emarginatus) turns into a pest fly eater around cattle
Source: PLoS One. 2024 May 8;19(5):e0302028. doi: 10.1371/journal.pone.0302028 (PMC11078406; doi:10.1371/journal.pone.0302028)
Supplement: S3 File — (PDF) [file pone.0302028.s014.pdf]

SUPPORTING INFORMATION

S3 File: List of references used to determine the pest status of Arthropoda prey.

[1–16]

1. Abbas N, Khan HAA, Shad SA. Cross-resistance, genetics, and realized heritability of resistance to fipronil in the house fly, *Musca domestica* (Diptera: Muscidae): a potential vector for disease transmission. Parasitol Res. 2014;113: 1343–1352. doi:10.1007/s00436-014-3773-4

2. Aizpurua O, Budinski I, Georgiakakis P, Gopalakrishnan S, Ibañez C, Mata V, et al. Agriculture shapes the trophic niche of a bat preying on multiple pest arthropods across Europe: Evidence from DNA metabarcoding. Mol Ecol. 2018;27: 815–825. doi:10.1111/mec.14474

3. Alford DV, Alford DV. Pests of fruit crops: a colour handbook. London: Manson; 2007.

4. Ancillotto L, Rummo R, Agostinetto G, Tommasi N, Garonna AP, de Benedetta F, et al. Bats as suppressors of agroforestry pests in beech forests. For Ecol Manage. 2022;522: 120467. doi:10.1016/j.foreco.2022.120467

5. Baldacchino F, Muenworn V, Desquesnes M, Desoli F, Charoenviriyaphap T, Duvallet G. Transmission of pathogens by *Stomoxys* flies (Diptera, Muscidae): a review. Parasite. 2013;20: 26. doi:10.1051/parasite/2013026

6. Baroja U, Garin I, Aihartza J, Arrizabalaga-Escudero A, Vallejo N, Aldasoro M, et al. Pest consumption in a vineyard system by the lesser horseshoe bat (*Rhinolophus hipposideros*). Jacobs DS, editor. PLoS ONE. 2019;14: e0219265. doi:10.1371/journal.pone.0219265

7. Baroja U, Garin I, Vallejo N, Aihartza J, Rebelo H, Goiti U. Bats actively track and prey on grape pest populations. Ecol Indic. 2021;126: 107718. doi:10.1016/j.ecolind.2021.107718

8. Mata VA, da Silva LP, Veríssimo J, Horta P, Raposeira H, McCracken GF, et al. Combining DNA metabarcoding and ecological networks to inform conservation biocontrol by small vertebrate predators. Ecol Appl. 2021;31: e02457. doi:10.1002/eap.2457

9. Pfiester M, Kaufman PE. Drone fly, rat-tailed maggot *Eristalis tenax* (Linnaeus) (Insecta: Diptera: Syrphidae). In: yumpu.com [Internet]. Feb 2012 [cited 29 Aug 2023]. Available: https://www.yumpu.com/en/document/read/12070625/drone-fly-rat-tailed-maggot-eristalis-tenax-edis-university-of-florida

10. Puig-Montserrat X, Flaquer C, Gómez-Aguilera N, Burgas A, Mas M, Tuneu C, et al. Bats actively prey on mosquitoes and other deleterious insects in rice paddies: Potential impact on human health and agriculture. Pest Manag Sci. 2020;76: 3759–3769. doi:10.1002/ps.5925

11. Van Emden HF. Handbook of agricultural entomology. Hoboken, NJ: Wiley-Blackwell; 2013.

12. Joint Research Centre (European Commission), Houston Durrant T, De Rigo D, Mauri A, Caudullo G, San-Miguel-Ayanz J. European atlas of forest tree species. LU: Publications Office of the European Union; 2016. Available: https://data.europa.eu/doi/10.2788/4251

13. Pest and disease resources. In: Forest Research [Internet]. [cited 15 Jun 2023]. Available: https://www.forestresearch.gov.uk/tools-and-resources/fthr/pest-and-disease-resources/

14. Atlas of Forest Pests. In: Forest pests Europe [Internet]. 2023 [cited 15 Jun 2023]. Available: https://www.forestpests.eu/

15. Gnaneswaran R, Wijayagunasekara HNP. Survey and identification of insect pests of oyster mushroom (*Pleurotus ostreatus*) cultures in central province of Sri Lanka. Trop Agric Res Ext. 1999;2: 21–25.

16. Hottel BA, Spencer JL, Ratcliffe ST. Trapping *Drosophila repleta* (Diptera: Drosophilidae) using Color and Volatiles. Fla Entomol. 2015;98: 272–275. doi:10.1653/024.098.0144
