# Supplementary material for: All-you-can-eat buffet: A spider-specialized bat species (Myotis emarginatus) turns into a pest fly eater around cattle
Source: PLoS One. 2024 May 8;19(5):e0302028. doi: 10.1371/journal.pone.0302028 (PMC11078406; doi:10.1371/journal.pone.0302028)
Supplement: S4 File — (PDF) [file pone.0302028.s015.pdf]

S4 File: Results of the variations in diet according to the locality variable

Curves of Hill numbers (q = 0 for prey richness computation)

None of the richness extrapolation curves reached the plateau for any localities. These curves suggested that, depending on the localities, between 47% (Durbuy) and 63% (Freyr) of the potential prey richness was recovered. In fact, as few individuals composed the Durbuy colony (11 bats counted maximum), collecting more guano was not an option. Therefore, we kept this site in all analyses while being cautious in the interpretations. In addition, graphics showed that confidence intervals (95%) partly overlapped between all localities, implying that the prey taxa diversity did not differ significantly spatially (Fig 1).

Fig 1. Accumulation curves of taxa species diversity. Alpha diversity was based on Hill numbers calculated for q = 0, corresponding to prey richness. Curves are drawn for each sampling locality and according to the number of guano bat samples analyzed for their diet. Shape symbols represent the observed values and dashed lines the extrapolated values expected with increased sampling effort. Shaded areas represent 95% confidence intervals. The observed percentage of sampled richness (observed value/estimated value*100) was: 56.78% (Aubel), 55.48% (Aulne), 46.65% (Durbuy), 63.40% (Freyr) and 51.40% (Rochefort). In the legend, n = number of individual fecal pellets.

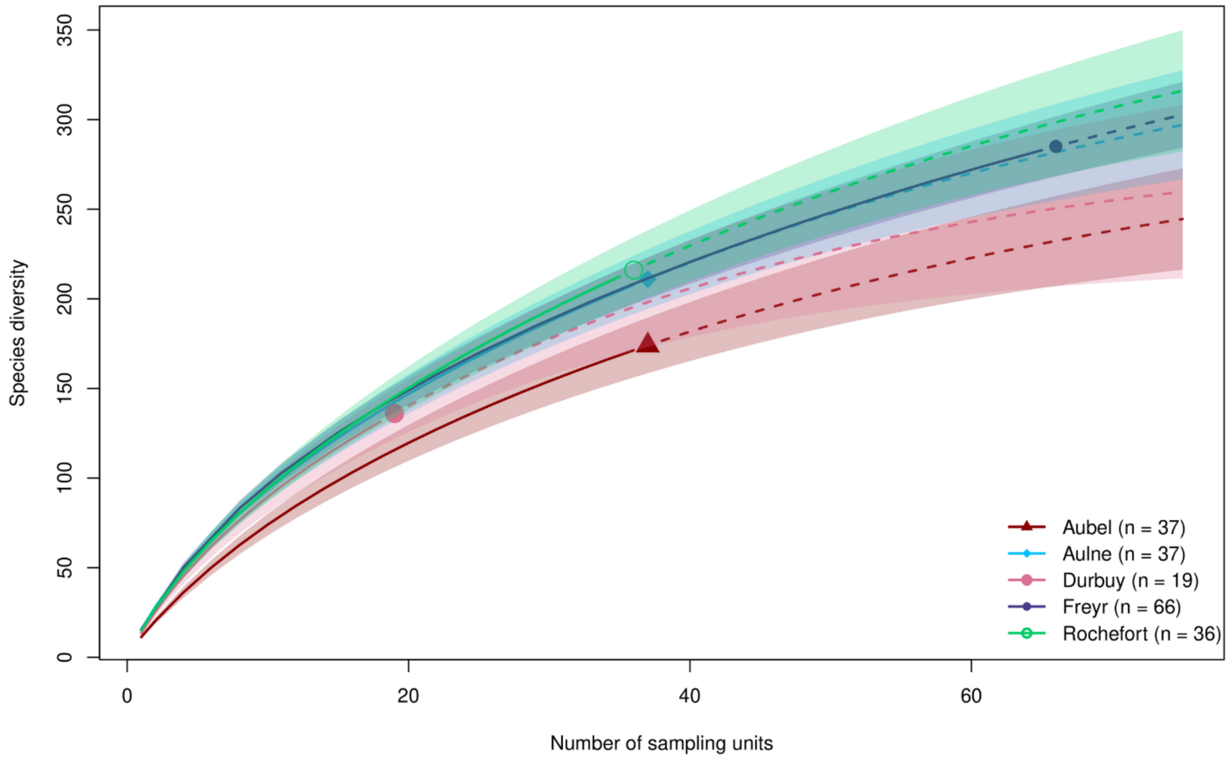

GLMM

When assessing the impact of the locality on the prey richness at the order level, all confidence intervals were large and overlapping (Fig 2). Yet some significant differences appeared between the localities regarding the order of Araneae and Hymenoptera (Table 1). Individuals from Aubel were characterized by a lower diversity of eaten Araneae than in other sites, while the opposite was observed in Rochefort colony (Fig 2a, Table 1). The main trend for the bats from Aulne colony was a larger diversity of consumed Hymenoptera taxa than in other sites (Fig 2e, Table 1). As regards Diptera, Lepidoptera, Coleoptera and Hemiptera consumption, no significant difference appeared (Figs 2b-2d and 2f).

Fig 2. GLMM visual outputs. Probability estimates (with 95% confidence intervals) of the taxa richness eaten by *Myotis emarginatus* per sampling locality and for the most consumed orders, as inferred by generalized linear mixed models with a quasi-Poisson distribution (glmmPQL function; equation: Order ~ locality + (1 | session)).

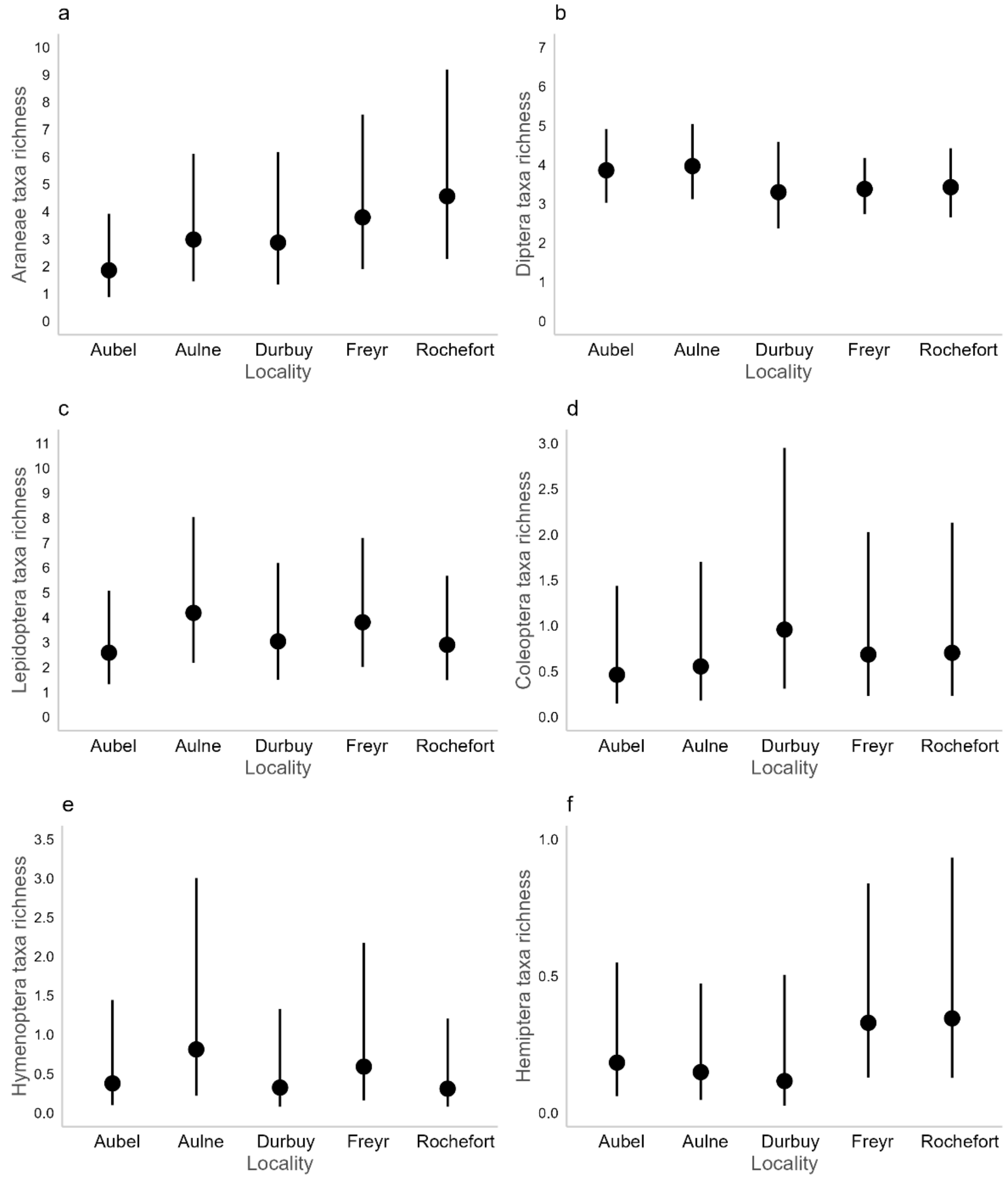

Table 1. GLMM outputs of the localities pairwise comparisons.

Fixed effect	*Estimate*	*Std.Error*	*z-value*	*P-level*	*Stat. Difference*	*Order*
Freyr-Aubel	0.713	0.200	3.587	< 0.003	Freyr > Aubel	Araneae
Rochefort-Aubel	0.898	0.210	4.279	< 0.001	Rochefort > Aubel	Araneae
Aulne-Aubel	0.766	0.253	3.024	0.020	Aulne > Aubel	Hymenoptera
Durbuy-Aulne	-0.918	0.338	-2.715	0.049	Durbuy < Aulne	Hymenoptera
Rochefort-Aulne	-0.963	0.278	-3.470	0.004	Rochefort < Aulne	Hymenoptera

Parameter estimates (quasi-Poisson distribution) of the localities pairwise comparisons calculated from the generalized linear mixed models (glmmPQL function; equation Order ~ locality + (1 | session)). Only significant effects are shown. Std.Error: Standard error).

PERMANOVA & PERMDISP

At the species level, PERMANOVA multivariate tests revealed a different taxonomic composition across sampling localities (df = 4, $R^2 = 0.036$, F = 1.781, $p < 0.001$) but only six pairwise comparisons were significant (Table 2). PERMDISP tests, which account for within-group variability to assess the group variances homogeneity, were non-significant for the locality variable (df = 4, F = 2.142, p = 0.080).

At the order level, compositional differences remained significant across localities (df = 4, $R^2 = 0.047$, F = 2.367, p = 0.009) but only four pairwise comparisons remained significant (Table 2). PERMDISP tests showed that within localities dissimilarity remained non-significant (df = 4, F = 1.754, p = 0.131).

Table 2. PERMANOVA outputs of the localities pairwise comparisons.

Pairwise comparison	***F-statistic (F)***	***R^2***	***Degrees of freedom (df)***	***p-value (p)***
At the species level				
Aubel-Aulne	F = 1.462	$R^2 = 0.020$	df = 1	p = 0.035
Aubel-Durbuy	F = 1.716	$R^2 = 0.031$	df = 1	p = 0.016
Aubel-Freyr	F = 2.297	$R^2 = 0.022$	df = 1	p = 0.002
Aubel-Rochefort	F = 2.881	$R^2 = 0.039$	df = 1	p = 0.001
Aulne-Rochefort	F = 2.111	$R^2 = 0.029$	df = 1	p = 0.004
Freyr-Rochefort	F = 1.948	$R^2 = 0.019$	df = 1	p = 0.010
At the order level				
Aubel-Aulne	F = 3.236	$R^2 = 0.043$	df = 1	p = 0.023
Aubel-Freyr	F = 2.842	$R^2 = 0.027$	df = 1	p = 0.047
Aubel-Rochefort	F = 5.789	$R^2 = 0.075$	df = 1	p = 0.006
Aulne-Rochefort	F = 4.375	$R^2 = 0.058$	df = 1	p = 0.012

Multivariate tests outputs of the localities pairwise comparisons of *Myotis emarginatus* diet composition at the species level (using Jaccard's dissimilarity index for presence absence data) and at the order level (using Bray-Curtis dissimilarity index for wPO – weighted percentage of occurrence data: within each sample, it is the prey item occurrence/total number of occurrences of all prey*100). Only significant effects are shown.

SIMPER

Overall, the diet composition dissimilarity between individual fecal pellets averaged 82.3% according to the SIMPER analysis. All locality pairwise comparisons including Aubel and Rochefort concerned, respectively, Diptera and Araneae, while Aulne was characterized by differences in Lepidoptera consumption (S5 Table).
