# Supplementary material for: All-you-can-eat buffet: A spider-specialized bat species (Myotis emarginatus) turns into a pest fly eater around cattle
Source: PLoS One. 2024 May 8;19(5):e0302028. doi: 10.1371/journal.pone.0302028 (PMC11078406; doi:10.1371/journal.pone.0302028)
Supplement: S5 File — (PDF) [file pone.0302028.s016.pdf]

S5 File: Niche overlap calculation using the Morisita-Horn index.

Methods: We calculated trophic niche overlap between samples originating from different sessions/localities via the Morisita-Horn index [1,2], which ranges from 0 (no overlap in resource use) to 1 (full overlap), using the mh function from the divo package [3].

Results: Regarding niche overlap between bats from different sessions, the Morisita-Horn index was the highest between July and August (mh = 0.86), while it was < 0.25 for June-July and June-August comparisons. Niche overlap between individuals from distinct colonies was the highest between Aulne and Freyr (mh = 0.82) but globally all pairwise associations remained high (mh > 0.60; Table 1).

Table 1. Morisita-Horn index.

	AUBEL	AULNE	DURBUY	FREYR	ROCHEFORT
**AUBEL**	1				
**AULNE**	0.785	1			
**DURBUY**	0.691	0.694	1		
**FREYR**	0.817	0.796	0.691	1	
**ROCHEFORT**	0.673	0.713	0.634	0.697	1

Niche overlap values for each pairwise locality association, calculated by the Morisita-Horn index. It ranged between 0 (no overlap) to 1 (full overlap) in prey taxa between the considered sampled localities.

References

1. Horn HS. Measurement of" overlap" in comparative ecological studies. Am Nat. 1966;100: 419–424.

2. Morisita M. Measuring of the dispersion of individuals and analysis of the distributional patterns. Mem Fac Sci Kyushu Univ, Ser E (Biol). 1959;2: 215–235.

3. Sadee C, Pietrzak M, Seweryn M, Wang C, Rempala G. divo: tools for analysis of diversity and similarity in biological systems. 2019. Available: https://CRAN.R-project.org/package=divo
