# Supplementary material for: All-you-can-eat buffet: A spider-specialized bat species (Myotis emarginatus) turns into a pest fly eater around cattle
Source: PLoS One. 2024 May 8;19(5):e0302028. doi: 10.1371/journal.pone.0302028 (PMC11078406; doi:10.1371/journal.pone.0302028)
Supplement: Attachment — Submitted filename: Response to reviewers.pdf [file pone.0302028.s017.pdf]

PONE-D-23-33429 Chloé Vescera

All-you-can-eat buffet: A spider-specialized bat species (*Myotis emarginatus*) turns into a pest fly eater around cattle

Editor answer:

Dear Dr. Vescera,

Thank you for submitting your manuscript to PLOS ONE. After careful consideration, we feel that it has merit but does not fully meet PLOS ONE's publication criteria as it currently stands. Therefore, we invite you to submit a revised version of the manuscript that addresses the points raised during the review process.

Please submit your revised manuscript by Feb 09 2024 11:59PM. If you will need more time than this to complete your revisions, please reply to this message or contact the journal office at plosone@plos.org. When you're ready to submit your revision, log on to https://www.editorialmanager.com/pone/ and select the 'Submissions Needing Revision' folder to locate your manuscript file.

Please include the following items when submitting your revised manuscript:

- A rebuttal letter that responds to each point raised by the academic editor and reviewer(s). You should upload this letter as a separate file labeled 'Response to Reviewers'.
- A marked-up copy of your manuscript that highlights changes made to the original version. You should upload this as a separate file labeled 'Revised Manuscript with Track Changes'.
- An unmarked version of your revised paper without tracked changes. You should upload this as a separate file labeled 'Manuscript'.

If you would like to make changes to your financial disclosure, please include your updated statement in your cover letter. Guidelines for resubmitting your figure files are available below the reviewer comments at the end of this letter.

If applicable, we recommend that you deposit your laboratory protocols in protocols.io to enhance the reproducibility of your results. Protocols.io assigns your protocol its own identifier (DOI) so that it can be cited independently in the future. For instructions see: https://journals.plos.org/plosone/s/submission-guidelines#loc-laboratory-protocols. Additionally, PLOS ONE offers an option for publishing peer-reviewed Lab Protocol articles, which describe protocols hosted on protocols.io. Read more information on sharing protocols at https://plos.org/protocols?utm_medium=editorial-email&utm_source=authorletters&utm_campaign=protocols.

We look forward to receiving your revised manuscript.

Kind regards,

Camille Lebarbenchon

Academic Editor

PLOS ONE

Dear Dr. Camille Lebarbenchon,

Thank you very much for having considered this manuscript for review and for the selection of skillful reviewers. They all made relevant comments which hopefully helped me bring this work to the next level. I took into account most of their wise suggestions to improve this manuscript in terms of language, scientific accurateness and conciseness. Thank you also for the advice you made to deposit my protocols on a designed platform. I already thoroughly described my methods in the manuscript and using supporting information. Any reader should therefore find all necessary experimental details in the present submission. However, I will consider the opportunity provided by PLOS ONE for publishing peer-reviewed Lab Protocol articles.

After having addressed all points raised during the review process, I now feel optimistic about the quality of this improved manuscript and about its compliance with the PLOS ONE's publication criteria.

Thank you again for the time devoted to the submission of this research.

Best regards,

Chloé Vescera

Journal Requirements:

When submitting your revision, we need you to address these additional requirements.

1. Please ensure that your manuscript meets PLOS ONE's style requirements, including those for file naming. The PLOS ONE style templates can be found at
https://journals.plos.org/plosone/s/file?id=wjVg/PLOSOne_formatting_sample_main_body.pdf and
https://journals.plos.org/plosone/s/file?id=ba62/PLOSOne_formatting_sample_title_authors_affiliations.pdf

➔ I double-checked all file names so that they meet PLOS ONE's style requirements.

2. We note that the grant information you provided in the 'Funding Information' and 'Financial Disclosure' sections do not match.

When you resubmit, please ensure that you provide the correct grant numbers for the awards you received for your study in the 'Funding Information' section.

➔ I modified it accordingly.

3. We note that you have referenced (Brinkmann, unpublished) on page 29, which has currently not yet been accepted for publication. Please remove this from your References and amend this to state in the body of your manuscript: (ie "Bewick et al. [Unpublished]") as detailed online in our guide for authors
http://journals.plos.org/plosone/s/submission-guidelines#loc-reference-style

➔ I formatted this reference such as detailed in the guide for authors.

4. We note that [Figure 1] in your submission contain [map/satellite] images which may be copyrighted. All PLOS content is published under the Creative Commons Attribution License (CC BY 4.0), which means that the manuscript, images, and Supporting Information files will be freely available online, and any third party is permitted to access, download, copy,

distribute, and use these materials in any way, even commercially, with proper attribution. For these reasons, we cannot publish previously copyrighted maps or satellite images created using proprietary data, such as Google software (Google Maps, Street View, and Earth). For more information, see our copyright guidelines: http://journals.plos.org/plosone/s/licenses-and-copyright.

We require you to either (1) present written permission from the copyright holder to publish these figures specifically under the CC BY 4.0 license, or (2) remove the figures from your submission:

a. You may seek permission from the original copyright holder of Figure 1 to publish the content specifically under the CC BY 4.0 license.

We recommend that you contact the original copyright holder with the Content Permission Form (http://journals.plos.org/plosone/s/file?id=7c09/content-permission-form.pdf) and the following text:
"I request permission for the open-access journal PLOS ONE to publish XXX under the Creative Commons Attribution License (CCAL) CC BY 4.0 (http://creativecommons.org/licenses/by/4.0/). Please be aware that this license allows unrestricted use and distribution, even commercially, by third parties. Please reply and provide explicit written permission to publish XXX under a CC BY license and complete the attached form."

Please upload the completed Content Permission Form or other proof of granted permissions as an "Other" file with your submission.

In the figure caption of the copyrighted figure, please include the following text: "Reprinted from [ref] under a CC BY license, with permission from [name of publisher], original copyright [original copyright year]."

b. If you are unable to obtain permission from the original copyright holder to publish these figures under the CC BY 4.0 license or if the copyright holder's requirements are incompatible with the CC BY 4.0 license, please either i) remove the figure or ii) supply a replacement figure that complies with the CC BY 4.0 license. Please check copyright information on all replacement figures and update the figure caption with source information. If applicable, please specify in the figure caption text when a figure is similar but not identical to the original image and is therefore for illustrative purposes only.
The following resources for replacing copyrighted map figures may be helpful:

USGS National Map Viewer (public domain): http://viewer.nationalmap.gov/viewer/
The Gateway to Astronaut Photography of Earth (public domain): http://eol.jsc.nasa.gov/sseop/clickmap/
Maps at the CIA (public domain): https://www.cia.gov/library/publications/the-world-factbook/index.html and https://www.cia.gov/library/publications/cia-maps-publications/index.html
NASA Earth Observatory (public domain): http://earthobservatory.nasa.gov/
Landsat: http://landsat.visibleearth.nasa.gov/
USGS EROS (Earth Resources Observatory and Science (EROS) Center) (public domain): http://eros.usgs.gov/#
Natural Earth (public domain): http://www.naturalearthdata.com/

➔ All maps displayed in this manuscript have been constructed using QGIS tools by the authors based on shapefiles freely accessible on the LifeWatch database (https://maps.elie.ucl.ac.be/lifewatch/ecotopes.html#). This platform offers quality maps and supports "Open e-Data for Biodiversity". This data complies with the CC BY 4.0 license which enables to:

- Share — copy and redistribute the material in any medium or format for any purpose, even commercially.
- Adapt — remix, transform, and build upon the material for any purpose, even commercially.
- ⇨ I therefore included the sentence mentioned above ("Reprinted from [ref] under a CC BY license, with permission from [name of publisher], original copyright [original copyright year].") in the figure caption but I did not complete the Content Permission Form as the original copyright holder explicitly states that the content is free to use.

Reviewers' comments:

Reviewer's Responses to Questions

Comments to the Author

1. Is the manuscript technically sound, and do the data support the conclusions?

The manuscript must describe a technically sound piece of scientific research with data that supports the conclusions. Experiments must have been conducted rigorously, with appropriate controls, replication, and sample sizes. The conclusions must be drawn appropriately based on the data presented.

Reviewer #1: Partly
Reviewer #2: No
Reviewer #3: Partly

---

2. Has the statistical analysis been performed appropriately and rigorously?

Reviewer #1: N/A
Reviewer #2: No
Reviewer #3: Yes

---

3. Have the authors made all data underlying the findings in their manuscript fully available?

The PLOS Data policy requires authors to make all data underlying the findings described in their manuscript fully available without restriction, with rare exception (please refer to the Data Availability Statement in the manuscript PDF file). The data should be provided as part of the manuscript or its supporting information, or deposited to a public repository. For example, in addition to summary statistics, the data points behind means, medians and variance measures should be available. If there are restrictions on publicly sharing data—e.g. participant privacy or use of data from a third party—those must be specified.

Reviewer #1: No
Reviewer #2: Yes
Reviewer #3: Yes

---

4. Is the manuscript presented in an intelligible fashion and written in standard English?

PLOS ONE does not copyedit accepted manuscripts, so the language in submitted articles must be clear, correct, and unambiguous. Any typographical or grammatical errors should be corrected at revision, so please note any specific errors here.

Reviewer #1: Yes
Reviewer #2: No
Reviewer #3: Yes

---

5. Review Comments to the Author

Please use the space provided to explain your answers to the questions above. You may also include additional comments for the author, including concerns about dual publication, research ethics, or publication ethics. (Please upload your review as an attachment if it exceeds 20,000 characters)

Reviewer #1:

Summary:
This study investigates the diet of the Geoffroy bat (*Myotis emarginatus*, ME) in several sites at different times during the breeding season. Fecal pellets were collected beneath roosting sites and analyzed with two COI metabarcoding that were further compared based on their respective detection success. The authors had several objectives: exploring the spatial and temporal variation of the diet and assessing the hunting strategy and foraging habitat used by ME by looking at prey ecological trait and habitat. The obtained results were also used to determine if the processed samples were a sufficient representation of the total Geoffroy bat diet.

General Comments:
I enjoyed this interesting work. The necessity to study the diet of Geoffroy bats in combination with the landscape is well explained. The methods are overall well described (there is some missing information, and some stats should be clarified), and most interpretations are supported by the results (in my opinion a few claims should be toned down in the Discussion). I also suggest including a section on secondary predation (e.g. it preys on spiders that prey on insects and/or other arthropods) in the discussion as it could have a big impact on diet richness estimation. Generally, I found the manuscript lengthy and sometimes hard to follow, so I believe the manuscript would benefit from being condensed a little bit (filler and unnecessary words could also be removed, e.g. of unnecessary words: "as a matter of fact").
Note: I haven't been able to access PRJNA1019357 (it was not found in SRA).

- ⇨ Thank you very much for this insightful and thorough review on my paper. These comments proved helpful to improve this work. As suggested, I added a section on secondary predation in the first part of the Discussion. I also removed some unnecessary sections, i.e. the ones that did not take part in the main story of this manuscript. I further refined this work by clarifying some stats and making explicit links between the goals, what has been done to achieve them and what were the final outputs and their interpretations. As such, I hope that the flow and clarity of the revised version have been improved.
- ⇨ Regarding the access to the sequence data related to this work, it will be allowed as soon as the paper will be released. This is why the given code is currently not working. However, I ensure you that raw sequencing data has well been deposited on this database. Here is a link created for reviewers, which provides read-only access to metadata: https://dataview.ncbi.nlm.nih.gov/object/PRJNA1019357?reviewer=lhpdp8f56thb7hjcnhr78iacs1. Note that this URL will expire when this BioProject is publicly-released.

Abstract

L46: You highlighted that ME prey on some pest species and could potentially be considered a pest regulator. We can't say from your data whether they have an impact on pest population as we just know that the species were detected in the feces.

- ⇨ I fully agree and modified it accordingly by replacing the concept of "regulator" by "consumer", the latter being only associated to the action of eating.

Introduction

L165-169: This section should not be in Introduction.

- ⇨ I removed this section and incorporated it in the Discussion.

Note: There is an arbitrary writing rule on using numerals for numbers 10 and greater and spelling out numbers one through nine.

- ⇨ I modified all number—except for numerals used for measurements—accordingly, thank you for the tip.

Material & Methods

L193: Why did you choose to refer to S1, S2, S3? I would recommend keeping the month instead of sessions (June/July/August or gestation/lactation/post-lactation). That would be so helpful to understand your data (linking your observations to the bat life cycle, energetic needs etc).

- ⇨ This indeed would be so much clearer. As such, I replaced each session by the corresponding month.

Figure 1: It is a pretty map, but as your paper focuses on landscape (forest, cowsheds) it would be more useful for us to know the landscape surrounding the sampling sites (forest, farm land etc.: CORINE land cover map?) instead of Wallonia regions.

⇨ Thank you for the advice, this would actually make much more sense in the context of this paper. I still kept this initial map to get a quick view of the location of the breeding sites in Wallonia but I placed it as supplementary material (S1 Fig). I then created two new representations: (1) a map depicting nine landcover classes within a 10 km radius around each bat maternity roost (Fig 1) and (2) a graph displaying the relative proportion of these landcover classes around each maternity roost (Fig 2). At the Walloon scale, we have a more precise land cover mapping than CORINE land cover map, i.e. the LifeWatch database (available here: https://maps.elie.ucl.ac.be/lifewatch/ecotopes.html).

L205: How long were they placed there? Indicate which decontaminant (e.g. bleach 20%).

⇨ I added the necessary details.

L206: What do you mean by proximity? A few cm, m? Do we have any information in the literature regarding ME individual location in their breeding sites? Do they stay to the same spot all the time? What about when individuals (adult-adult, adult-offspring/young) stay very close to each others? I believe ME can also make clusters. I'm not convinced by your assumption of 1 pellet = 1 individual and I believe this should be modified in the entire manuscript.

⇨ I entirely agree with this comment. My previous assumption that 1 pellet = 1 individual was in fact not rigorous as I did not individualize the samples. I therefore could not ensure that 2 pellets did not originate from the same individual. As consequence, I modified this term through the whole manuscript by replacing "individuals" by "individual pellets" or "samples". I also toned down the interpretations appropriately.

L229: This is indeed uncommon as using technical replicates is a golden standard. The fact that you used two markers is clearly not enough to justify the absence of replicates. It is expected that the two primer pairs won't give the same results (that's why you combine two markers) so you cannot use the absence of overlap between them to detect false positive (+ they have their own biases). You must rather emphasize here how you managed to decrease false positive detections using bioinformatics (denoising, chimera removal, etc) and further filtering (negative controls, min number of reads etc) in R/Excel. You filtering approaches must be conservative to compensate for the lack of technical replicability.

⇨ In fact, there was congruent identification for half and two-thirds of the prey taxa identified via Galan and Zeale primers, respectively, thereby boosting our data reliability. As mentioned by the reviewer, this was however not sufficient to bypass the use of technical replicates. I therefore wrote a paragraph describing how I countered this bias in the Methods section.

L232/241: Indicate the concentration of the Hot start polymerase.

⇨ I added this information.

L251: Indicate here or in the S1 File the percentage of PhiX that was used for the sequencing run.

⇨ I added this information in the S1 File, which contains additional information about the methods.

L265: And the coverage percentage? If the identity is 100% but the coverage is only 5%, then the taxonomic assignment is not good.

⇨ For the taxonomy assignment in QIIME 2, I used the classify-sklearn method relying on a Naive Bayes classifier which typically operates on sequence fragments rather than full-length reads. The concepts of identity and coverage are therefore not applicable to this technique. Instead, confidence values represent the raw probability estimated by the Naive Bayes classifier, i.e. the predicted probability that the assigned taxon is accurate.
Although confidence value does not directly correspond to percent identity, a high confidence score at a fine taxonomy level (e.g. species level) generally suggests a high percent identity database match. Nevertheless, a high percent identity match may also result in a low confidence, if the matched sequence is identical across different taxonomic groups.

L266: It's always amazing to see how good the Galan's primers are, with such a low % assigned to arthropods it still has a greater species coverage than arthropod specific primers like Zeale. Neat!

⇨ I cannot agree more! They are very useful when studying insectivorous bat diets!

L274: Did you keep strictly ME samples or also samples with a mix of both species? If the latter please justify here as it would have big consequences on your diet assessment.

⇨ I did not mention how I segregated the samples as the level of contamination was very low. Indeed, following strict sequence filtering, the contamination ratio from *M. emarginatus* to *R. ferrumequinum* or conversely reached 0.61% (134 reads of *M. emarginatus* and 22,092 reads of *R. ferrumequinum* in a sample from Orval) and 0.09% (166 reads of *R. ferrumequinum* and 186,214 reads of *M. emarginatus* in a sample from Freyr), respectively. I clarified this in the S1 File.

L275: How did you deal with redundancy or pseudogenes in your dataset? For example, when you both detected a taxa at the species and genus or family level in the same sample?

⇨ This information was indeed lacking and I thus added the following sentences in the supplementary S1 File: "To further deal with read redundancy, ASVs assigned to the same taxa and at the same taxonomic level were pooled." and in the main text: "Throughout this manuscript, the "taxa" term has been used to encompass several taxonomic levels, but always with the most precise identification achievable." (see lines 298-300).

L303: Not sure to get the sentence. You established or aimed at establishing whether *B. taurus* DNA was present in ME samples through secondary predation?

⇨ This indeed was not clear. In the reviewing process, I moved this topic further down in the M&M section and described it in more details: "As ME has been previously reported to feed in cowsheds [30,31,49], we noted when *Bos taurus* DNA was detected in the bat fecal pellets. Cow DNA was detectable by means of secondary predation, i.e. through bats feeding on flies—mostly *S. calcitrans* and *M. domestica*—themselves feeding on cattle blood [27,56]."

L309: Indicate here that species richness is equivalent to q = 0 for Hill numbers (q = 0 is also indicated in Figure 3b but not 3a)

⇨ I added this information in the S1 File, which contains additional information about the methods.

L310: "Working on sample coverage"? Please clarify what you did.

⇨ This analysis was ambiguous and unnecessary and I therefore removed it.

L313: I'm surprised that there wasn't any overdispersion of the data, have you checked?

⇨ I checked and there was overdispersion for most of the ran models. Thus, I decided to run quasi-Poisson models instead of strict Poisson models to offset this issue.

L315: Not clear in which case locality was a fixed effect, when session was a fixed effect and why it wasn't always the same variable as fixed/random effect. Could you clarify this section please?

⇨ The **Fixed** Effects are the predictors that have a fixed and constant effect on the response variable.
The **Random** Effects are additional sources of variability that are not fixed but are specific to certain groups or clusters in the data.

⇨ Here, I ran 2 types of models (but moved the results of those where "locality = fixed variable to the S4 File) depending on whether I considered the session/locality as the fixed or random effect.
  - Thus, when assessing the **impact of the 3 sessions** on the mean richness of prey at the order level, I also wanted to capture the variability between samples that was not explained by the session factor but which was attributed to the locality.
  - Conversely, when assessing the **impact of the 5 localities** on the mean richness of prey at the order level, I also wanted to capture the variability between samples that was not explained by the locality factor but which was attributed to the session.

L316: individual bats or individual pellet? See comment above on the assumption 1 pellet = 1 individual.

⇨ As recommended and to be more honest in relation to the available data, I removed any inferences about the number of individual bats.

L320: Have you checked the assumption of the PERMANOVA? It would be valuable to run a betadisper() alongside PERMANOVA, because a significant output might mean that homogeneity/heterogeneity of variances contribute to any observed differences in PERMANOVA.

⇨ Thank you for this advice! I complemented the PERMANOVA analyses by betadisper analyses as it could indeed have large consequences on the interpretations of the former.

L324: You mean for further analyses (IBD, NMDS)?

⇨ I meant for the SIMPER analysis only. It was indeed not clear and I therefore modified the words "For data analysis" into "For this analysis".

L331: Indicate how many try and trymax in metaMDS. Please also indicate the stress value on your NMDS figures as it indicates the quality of the solution: usually a stress values lower than 0.05 indicate that the solution is of excellent quality and stress values higher than 0.2 indicate solution of poor quality (Kruskal, 1964).

⇨ I added this information in the S1 File, which contains additional information about the methods.

L334: closed landscapes: do you mean forests or also other types of closed landscape?

⇨ To make this work clearer but also more compelling, I removed this analysis, which aimed to evaluate whether eaten spiders were associated to closed-landscapes (i.e. natural cluttered environments such as forests). Far from being uninteresting *per se*, this section did not exactly fit into the goals and I therefore preferred deleting it.

L343: Replace "determined" by "estimated"

⇨ I modified it accordingly.

L348: "We investigated the potential link between locality and caterpillar life stage with Kruskal-Wallis analyses" I don't understand what you tested here: for example, whether there more caterpillars at locality A than B? One test per category? (corrected for multiple test?)

⇨ The idea was initially to assess whether Lepidoptera taxa in the larval stage were found in similar proportion in all colonies. However, I preferred removing this analysis as it was not answering any hypothesis, hence disrupting the flow of the Methods section.

L359: I'm a little bit confused again with the stats here. Is Chi² good to use here? Why these two species get another test and on another dataset?

⇨ Again, I overloaded the Method section with numerous tests which were not always useful. As such, I cut out the following "To examine differences in the detection of *B. taurus* DNA among localities/sessions, we applied Chi-squared tests on the wPO dataset (chisq.test)." to only retain the Kruskal-Wallis test on the a subset wPO dataset. I also clarified the need to work on a subset of the wPO dataset restricted to the samples containing cow DNA in light of the secondary predation.

L364: Could other domestic (e.g. horses, sheep, donkey) and wild (e.g. deers) animals be important for flies?

⇨ Yes, they are. Patra et al. 2018 (see reference [27] in the manuscript) reviewed the importance of *Stomoxys calcitrans* in livestock and mentions this: "Among the domestic animals, horses, cattle, pigs, dogs, cats, sheep and goats appear to be most preferred host." However, these animals are:

- rarely aggregated in number as high as those observed in cattle farming (e.g. horses, donkey, cats, dogs and goats).
- rarely bred in Wallonia (e.g. only 3% of farms have pig breeding activities).
- rarely bred in some parts of Wallonia considered in this study.
- mainly locked in stables.

- ⇨ Deers [1] are also targeted by *Stomoxys* flies. However, feeding on these animals is hampered by the fact that they are mobile prey scattered through the forested landscapes (contrary to cattle).
- ⇨ Anyhow, no DNA from these species have been retrieved from our samples, even in small quantities.

<u>Results</u>

Figure2: text is too small / resolution not good enough. A tip: you can use “theme(axis.text.x = element_text(angle = 50, vjust = 1, hjust = 1))” to rotate a bit your arthropod name so they fit entirely (no need to shorten them).

- ⇨ Thank you for the tip, I modified it accordingly.

L391: Very interesting, did you also look at taxonomic resolution between the two markers? Is Galan or Zeale more resolutive than the other?

Zeale

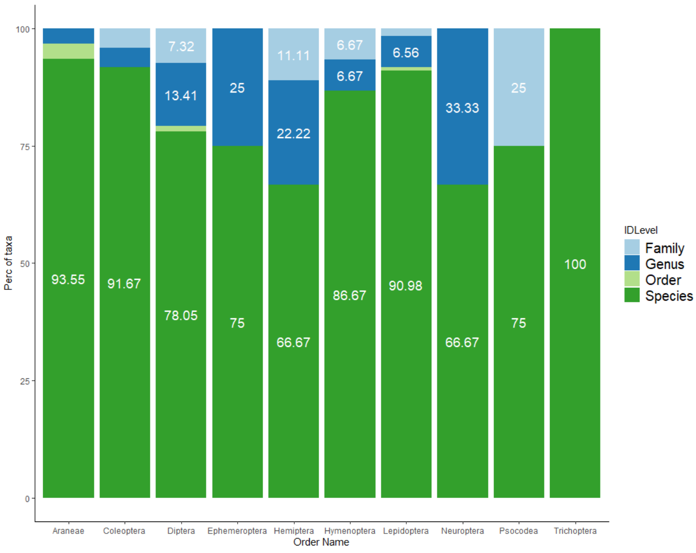

Galan

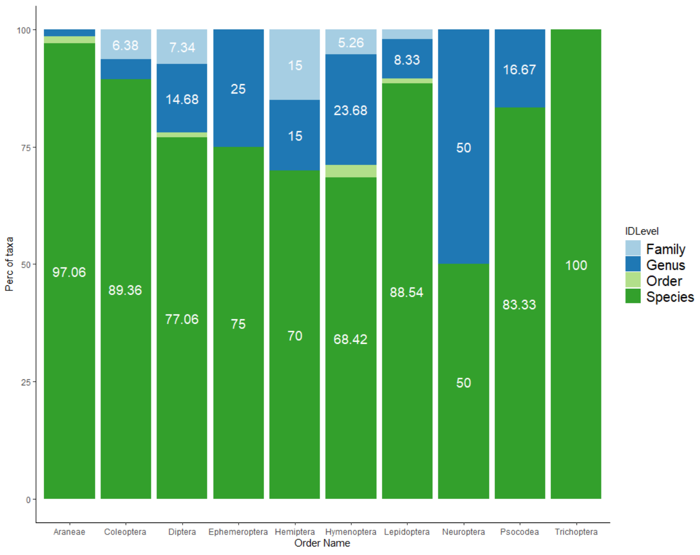

- ⇨ I checked the taxonomic resolution between the two markers and here are the output graphs for the 10 main taxonomic orders. As you can see, the resolution is very similar between both primer pairs and even reached 100% at the species level for the order of Trichoptera. Only for the Hymenoptera and the Neuroptera did the Zeale primers appear much more efficient than those from Galan, as the former primers achieved a species level resolution of 86.67 and 66.67%, respectively, instead of only 68.42 and 50%, respectively, for the latter. The only taxonomic order for which the Galan primers were more resolutive at the species level was the Pscocodea as it reached 83.33% of assignation against 75% for Zeale.

L402: you can remove “different”.

- ⇨ I deleted it.

L403: Very cool to see this pattern in ME too (most taxa are rare, i.e. occurring in one sample --- similar results were observed for the greater horseshoe bat; Tournayre et al. 2021. eDNA metabarcoding reveals a core and secondary diets of the greater horseshoe bat with strong spatio-temporal plasticity)

- ⇨ Thank you for mentioning this very interesting work! I included a sentence in the Discussion (see lines 653-656) to corroborate my findings in the light of the research on *R. ferrumequinum*.

L409: In this section you include some interpretations that don’t belong to a Results section (e.g. “highlighting few unsampled taxa at a temporal scale while sampling effort could have been improved spatially.”

- ⇨ I fully agree and modified it accordingly.

L435: if you use a function/package to retrieve and compare the contrast (Table 1, Fig 4), please indicate it in the Methods.

⇨ I added this information in the main text.

L469: you can put the betadisper results here

⇨ I added betadisper results just following the PERMANOVA results and I also detailed the meaning of both tests to help the reader to understand the implications of the results (see lines 681-683 and 688-689).

Page 19-20: formatting is weird here, probably when everything has been merged into a single pdf.

⇨ I am actually not sure of what is weird here. The table caption is just under the table itself and the caption text size is smaller than the main text. Maybe is that the weird thing? Or eventually is it the table formatting? Or perhaps the line counts which (wrongly) went on behind de table?

L489, 558: I'm not convinced by "individual bat" (see previous comments)

⇨ As recommended and to be more honest in relation to the available data, I removed any inferences about the number of individual bats.

L535: Please, tone down a little bit, for example by using "presumably" (very likely though not known for certain) eaten as caterpillar, imago etc

⇨ I added adjectives of nuance.

L553: Again it would be nice to see on your map the different forests to make the link with you're saying here.

⇨ As suggested, I created a map depicting nine landcover classes (Fig 1) and their relative proportion around each roost in a related graph (Fig 2).

L573 from S1, S2, and S3, respectively (respectively is usually at the end in english)

⇨ I modified this through the whole paper, thank you for the English revision.

L576: did not differ at all?

⇨ I replaced "largely" by "significantly" to be more accurate.

Discussion

L608: There has been another study published last Spring: See Vallejo et al. 2023 they studied five maternity colonies in the Basque Country every 2 weeks for the whole duration of the maternity season. Like you they found significant changes in diet diversity and composition but the opposite way "At the beginning of the season, *M. emarginatus* consumed a variety of prey orders, Diptera being the most abundant; but as the season progressed, the relative consumption of Araneae increased." I highly recommend you reading this paper to nourish your discussion.

⇨ Many thanks for this suggestion. I carefully read this interesting work and discussed about our distinct results (see lines 761-771).

L637: The Galan and Zeale target fragments highly overlap so I doubt the reason is new sequences in databases. I rather think that Zeale used to be the most commonly used in insectivorous diet studies so most people were sticking with it without trying to get better, and now you and others are showing that minibarcodes with degenerate primers such as Galan's can do much better. It's interesting because this questions what we use as standards and how that has biased our knowledge on bat diet.

⇨ Thank you for this relevant comment. I included additional references to support the recent use of Galan's pair of primers in the study of invertebrate and vertebrate diets.

L640-644: I agree: it amplifies the predator DNA and yet it still detects more arthropod than Zeale so I don't think it is too much of an issue (+ in Tournayre et al. 2020 they showed that the percentage of bat reads had no effect on the number of

occurrences detected). Would be interesting that you test it with your data to see if you get the same signal or not (although probably less stat power as it would be just galan vs zeale…?).

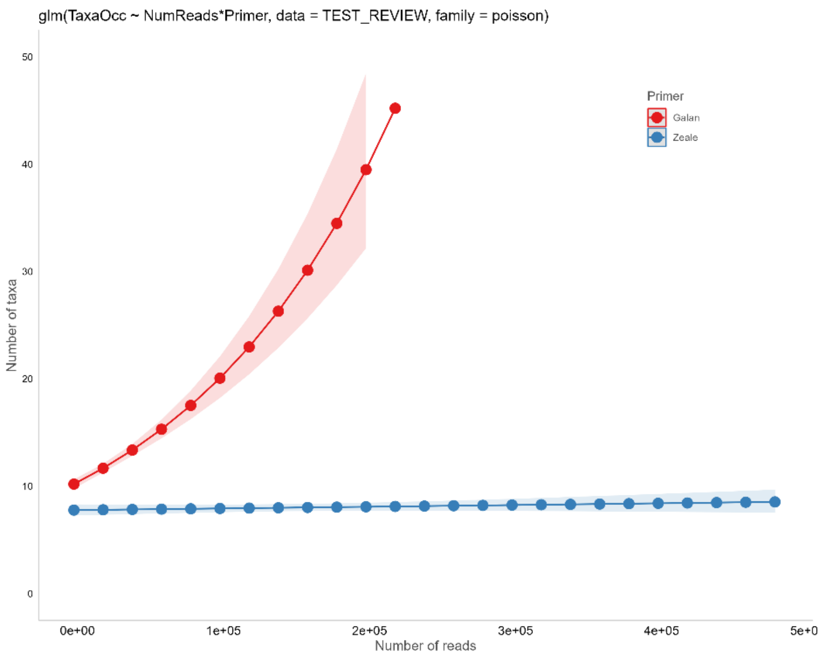

⇨ To move towards conciseness, I removed this section from the paper but I still ran a glm model with a Poisson regression (lme4 package), similarly to what has been done in the study you mentioned.
As you can see on the graph on the left, the number of reads seemed to impact the number of final taxa only when using the primers from Galan, while the number of taxa remained even no matter the number of sequenced reads with those from Zeale. This steep rise for the Galan marker could indicate that taxa retrieval was not exhaustive. However, the graph shows that even the lowest number of taxa detected in samples with the Galan' primers is predicted to be above any number of taxa in the Zeale samples, hence highlighting the power of the degenerated Galan primers.

L651: here you could emphasize that it's very common (if not always the case?) in metabarcoding diet study; it's not that you did something wrong, it's just incredibly hard to reach the plateau.

⇨ Thank you for raising this point. I included some references supporting this general issue.

L662: how would you assess alterations in insect populations owing to anthropogenic pressures based solely on metabarcoding studies?

⇨ Metabarcoding studies on the diet of insectivorous bat species–if carried on over several years–could help assessing whether some arthropod species appear or disappear. Of course, these studies should always be complemented by field evaluations to attest of these arthropods' presence/absence. Yet metabarcoding tools could alleviate the field workload involved in answering such questions by identifying appearing/disappearing taxa and focusing the fieldwork on these specific insects. To moderate my claims, I included the temporal notion "if carried on over several years" in the sentence.

L668: add refs for the metabarcoding studies (e.g. Vallejo et al 2019, Vallejo et al 2023)

⇨ I added the references.

L686: presumably* prey

⇨ I added adjectives of nuance.

L716: It would be good to mention secondary predation issue somewhere (metabarcoding cannot distinguish the prey from the prey of the prey): it surely inflates diversity.

⇨ I added a whole paragraph explaining the concept of secondary predation and its implications in terms of data interpretation (see lines 657-675).

L735: I think it would be very valuable to make an analysis based on the landscape composition…

- ⇨ To move towards conciseness and clarity, I removed this section from the paper. Anyhow, as suggested and as mentioned earlier in this file, I created a map (Fig 1) depicting nine landcover classes and their relative proportion around each roost in a related graph (Fig 2).

L743: I don't know if I just misunderstood the sentence, but if not please be careful in your claims because you don't have prey availability data. It sounds like you're circling back your data here.

- ⇨ I surely acknowledge that I did not estimate the prey availability in the study area. However, I did calculate prey richness and how it varied along the breeding season at both the species (with Hill numbers) and the order (with GLMMs) levels. I therefore used this information and nothing more to explore the ecological reasons of the temporal shifts in preferred prey.

L747: suggests instead of indicates (again you don't have prey availability to support your claim)

- ⇨ Thank you for pointing out this overstatement. I modified the sentence accordingly.

L751: Any idea why it is the opposite pattern in the Basque country (Vallejo et al 2023)?

- ⇨ Instead of explaining the differences between our results and the ones from the Basque country (Vallejo et al. 2019 and 2023), I discussed about the reasons of the different results between Vallejo et al. 2019 and ours versus Vallejo et al. 2023 (see lines 763-769), because our results are similar to those from Vallejo et al. 2019. Vallejo et al. 2023 mentioned that opposite trends between their two studies could be due to the differences in foraging habitat availability.

L755: I know my comment is redundant but I'm not comfortable with the "two third of bat individuals", you don't know if your pellet belonged to one or several individuals - especially as adult individuals and mother/offspring can stay very close to each other. The latter would of course have an impact late summer when the young starts to hunt.

- ⇨ As recommended and to be more honest in relation to the available data, I removed any inferences about the number of individual bats.

L817: would be good to see on the map :)

- ⇨ As suggested and as mentioned earlier in this file, I created a map (Fig 1) and a related graphic depicting nine landcover classes and their relative proportion around each roost (Fig 2). I then used the results depicted in these representations to explain differences in the Lepidoptera habitats.

L881: And does ME also preys on endangered insect species? I so agree that bats are invaluable allies, but they probably also prey on endangered species. It would be nice if you could look into your data, especially as you are talking about how the decline of insect might affect ME in the Intro.

- ⇨ There is no doubt that *M. emarginatus* preys upon taxa rarely occurring in Wallonia and even in Belgium, as you can see in the S3 Table (see the taxa highlighted in red, orange and blue and the corresponding legend in the second Excel sheet). However, distinguishing endangered taxa from those featuring a natural sparse distribution in the study area is difficult. What should be noted however is the low taxa occurrence of each of these rare taxa in the *M. emarginatus* diet. Conversely, most taxa commonly occurring in its dietary spectrum (in > 30 samples) were commonly retrieved in Belgium according to the Observation.be platform (see the "status" column). Only *Araneus angulatus* is categorized as "rare" on the web portal Observation.be but it is categorized as "Least Concern" on the IUCN red list from France. Its consumption my *M. emarginatus* therefore should not be a concern for the viability of the spider species.

Table S1 and S2: Please complete the caption: "ME" = *Myotis emarginatus*, what are "CD" and "ED" column (I assume the codified date for analyses)? What's the difference between "NumberMEAdults" and colony size? Is colony size ME+RhiFer?

- ⇨ I described all abbreviations in the caption of the supplementary tables but they are also detailed in the last sheet of each Excel file. In addition, I also renamed the column "NumberMEAdults" into "NumberME" and modified its content accordingly (adults+juveniles *M. emarginatus*) as these column names were not clear.

Again Table S3: Please complete the caption: what do the Column names mean, same for color code (why some species are highlighted in blue, red, orange)

- ⇨ Because the caption would be lengthy by including the description of all abbreviations, I added this sentence to the caption "The legend of the column names and the color code are detailed in the second sheet of the file."

Table S4: Please complete the caption: color code?

- ⇨ Because the caption would be lengthy by including the description of all abbreviations, I added this sentence to the caption "The legend of the column names and the color code are detailed in the second sheet of the file."

Cited literature:

1. Pospischil R. The stable fly *Stomoxys calcitrans* (Linnaeus 1758) (Diptera: Muscidae) – biology and impact on livestock. 2012 [cited 1 Feb 2024]. Available: https://www.academia.edu/10810969/The_stable_fly_Stomoxys_calcitrans_Linnaeus_1758_Diptera_Muscidae_biology_and_impact_on_livestock

Reviewer #2:

Major comments:

Vescera et al. presents a study that describes the spatio-temporal variation in the diet of *M. emarginatus*, and then makes inferences about foraging behavior from bat diet. The laboratory techniques used for this study seem to be very thorough and I think using life history data from prey species to infer predator behaviors is fascinating! However, I do have some serious concerns about the authors' overextension of their results and, by association, some of the major claims of their paper.

First, I think the emphasis on spatial variation is a bit overplayed. According to IUCN, this species ranges across most of Europe and parts of Africa and Asia. Your field sites seem to be an average of 50-75 km apart and only exist in their northern range. This to me is not spatially varied, especially as no information is provided in the Methods that discusses the variations in the landscapes at each of these data points (this is not seen until the Discussion). Focusing on the novelty of your work as the northern-most extreme for this species would be more accurate – which you somewhat allude to in Lines 712-714.

- ⇨ Thank you very much for raising this point. I fully agree with the reviewer that the five colonies are too close to each other to talk about spatial variation. As consequence, I narrowed the topic to exploring the diet at the northern edge of the species range, where this thermophilic bat would probably encounter ecological limits.

Second, temporal variation typically represents several time points throughout the year or life stages of an animal. With hibernating bats, the yearly active period is truncated in parts of the world due to hibernation, which can limit the number of time points to look at diet. However, bats are active on the landscape outside of the breeding season of June-August that has been identified here in this study. Therefore, to accurately assess temporal variation, bat diet should also be sampled outside of the breeding season if a study aims to look at actual temporal variation in bat diet. This study only focused on three time points during the breeding season (although even the temporal "sessions" are not explicitly defined, only in the Discussion does it say that each are explicitly a single month and align with specific periods of the breeding season). Additionally, this study only collected one season of data, which is not an accurate metric for temporal variation. I think it is an overstatement that this study looks at temporal variation and instead should narrow their inference to breeding season variation. Still incredibly important and is also more accurate given the presented data.

- ⇨ Again, I could not agree more. At first, I clearly defined the temporal limits of the sessions and further renamed them according to the associated month (i.e. "June", "July" and "August") instead of using unnecessary abbreviations (i.e. "S1", "S2" and "S3"). Then I reframed the goal by stating that I would investigate the variations of the diet throughout the breeding season. Yet I kept using the term "temporal" sparsely through the manuscript as the breeding season is still connected with and limited by time, no matter the considered temporal scale.

Third, the authors use previously published literature on prey species behaviors to try and characterize the foraging and prey hunting patterns of *M. emarginatus*. I think the idea behind this is really impressive and innovative, and I applaud the authors' efforts. However, I think the authors need to measure their claims a bit about where bats are foraging. Explicitly stating that bats traveled and foraged at cowsheds, when your only evidence for this is that they consumed flies that contained DNA of cows, is extreme as the authors do not provide any additional evidence from this study to support their claim. How do the authors know that bats are foraging on these insects from cowsheds? From scanning the literature, flies can disperse several kilometers from their origin, meaning that bats may not be necessarily taking these pests from cowsheds (Nazni et al. 2005, Kjærsgaard et al. 2015). Therefore, some serious alterations in the language used to describe this potential relationship are needed. I recommend removing the focus on cowsheds from the title as well unless you introduce evidence that definitely shows bats are in cowsheds (like telemetry data, acoustic data, or capture data).

- ⇨ This is true that I have overplayed the whole story by attesting that *M. emarginatus* bats foraged in cowsheds. Thus, I reformulated this term throughout the whole manuscript and instead talked about "cowsheds and their surroundings" or "cowsheds and the surrounding pastures". Anyhow, in respect to the hunting style of *M. emarginatus*–which is mostly a gleaner species, I would assume that most flies were effectively caught in cowsheds. As consequence, I also removed the focus on cowsheds in the title and instead mentioned that the bats occurred around cattle. Regarding the Lepidoptera stages which are inferred from a bibliographic search, I also added nuance to my statements by including words like "presumably" and "likely".

Lastly, throughout the manuscript, the authors refer to their samples as collected from individual bats. While the methods state that efforts were made to collect feces some distance apart so that it was likely from another bat, bats alter position in the roost all the time so there is no way that this method confirms data collection from individuals. If there was genetic analysis done to show that fecal samples were confirmed to be from individuals, that information needs to be included in the

main manuscript. As such, language and inference needs to be adjusted to reflect that samples are from colonies and not individuals, so behavioral inferences can only be made on a colony level, not the individual.

- ⇨ Other reviewers also mentioned this overstatement and I entirely acknowledge that I cannot ensure that 1 pellet = 1 individual. As consequence, I replaced the term "individuals" through the whole manuscript by "individual pellets" or "samples". I also toned down the interpretations appropriately.

Given these concerns, I suggest that the authors narrow the narrative for this manuscript to looking at breeding season variation in the northernmost point of the range of *M. emarginatus*. I think that is a more accurate representation of the results and is also still compelling. In the discussion, the authors could then provide inference about the foraging ecology of this species based on the diet, without stating that their study shows that bats are foraging in specific areas and using particular hunting strategies (because your study does not show these things). A very similar study recently published by Vallejo et al. in Mammalian Biology did a great job of not over-extending their results but still making inferences about foraging; Vescera et al. could do something similar that still shows how cool this information is without overdoing it. As a general note, the grammar and language in this manuscript needs to be reviewed for conciseness, correctness, and for confusing sentence structure. In the line comments, I have highlighted areas I noticed that need assistance. However, I recommend the authors closely proofread their revision or ask for assistance with proofreading the English used in this manuscript in future versions.

- ⇨ Thank you for these valuable comments on my manuscript. They revealed very helpful and they brought my work to the next level. I asked assistance to proofread the language and did an intense work of cutting-out unnecessary paragraphs/words and fillers to move towards clarity and conciseness. I also carefully read the very interesting study from Vallejo et al. (I apparently missed their work from 2023 so thank you for mentioning it!).

Cited literature:

Kjærsgaard, A., Blanckenhorn, W. U., Pertoldi, C., Loeschcke, V., Kaufmann, C., Hald, B., ... & Bahrndorff, S. (2015). Plasticity in behavioural responses and resistance to temperature stress in *Musca domestica*. Animal Behaviour, 99, 123-130.

Nazni, W. A., Luke, H., Rozita, W. W., Abdullah, A. G., Sa'diyah, I., Azahari, A. H., ... & Sofian-Azirun, M. (2005). Determination of the flight range and dispersal of the house fly, *Musca domestica* (L.) using mark release recapture technique. Tropical biomedicine, 22(1), 53-61.

Vallejo, N., Aihartza, J., Olasagasti, L., Aldasoro, M., Goiti, U., & Garin, I. (2023). Seasonal shift in the diet of the notched-eared bat (*Myotis emarginatus*) in the Basque Country: from flies to spiders. Mammalian Biology, 1-13.

Line comments:

Abstract

Line 27: reword to "…conservation, as it will aid our understanding of foraging behavior plasticity in response to reduced insect populations." The way it is currently worded is cumbersome and could be refined to make your point clearer.

- ⇨ I modified it accordingly.

Line 28: reword to "Despite the global decline in insects,…" so it is more of standalone thought

- ⇨ I modified it accordingly.

Line 29: The English in the first part of this sentence needs a little work. Try something like "Past research has bypassed the potential of European bats to regulate such pest populations, despite their economic relevance." This will also be important to adjust as there is a lot of work looking at pests in bat diet, so you will need to fix this sentence to make it more specific to your system.

- ⇨ I partially followed this advice. I indeed replaced "regulate" by "suppress" as studying the bat regulation of insect populations would require an experimental field study (e.g. a study involving bat-excluded plots and control plots), which is out of scope in this work.

Line 30: Remove “thus”.

⇨ I modified it accordingly.

Line 31: add “the” before “diet composition”.

⇨ I modified it as suggested.

Line 31: See my major comments above - you didn’t actually look at foraging behavior or pest hunting – you looked at diet and spatio-temporal variation. Adjust this sentence to show what your study actually focused on.

⇨ I rewrote the aims of the study more accurately according to the methods.

Line 32: Would you say *M. emarginatus* is a habitat specialist? Try to streamline this part of the sentence: “of a bat species originally favoring closed or semi-open habitats”, as it’s quite chunky and starts to seem like a run-on sentence.

⇨ I removed the mention about which habitat is preferred.

Line 38: There is no data presented in this manuscript placing bats at cowsheds. Rephrase to say that they may be using cowsheds

⇨ I instead talked about cattle farming.

Line 41: rephrase “~50% of taxa occurrences concerned these flies.” Do you mean “these flies accounted for almost 50% of Dipteran occurrences”? Rephrase to make it more interpretable.

⇨ I rephrased the sentence as suggested.

Line 42: Are you inferring that bats are switching their diet in the middle of a single year due to decreases in insect populations? You do not provide any evidence to support this claim and the data you do provide are not comprehensive enough to support this statement. Revise to something more relevant to the data you present, maybe along the lines of breeding season needs this far north or something.

⇨ I acknowledge that this sentence might be confusing. I do not mean that bats are switching their diet in the middle of a single year due to decreases in insect populations, but instead that the plasticity in foraging behaviour displayed by *M. emarginatus* bats could be very beneficial seeing the ongoing global drop in insect densities. As such, we have seen that this species was able to exploit food from diverse foraging sites, including anthropic habitats. Although your suggestion to reformulate this sentence in the light of the breeding season needs under the suboptimal conditions that may apply at the northern edge of a species range is relevant, I preferred sticking to the idea of “dropping insect populations” as it really was the central information around which all my objectives were structured. However, I removed the focus on “ecological traps” to make the sentence more concise and impactful.

Line 45: This sentence seems like a random addition and comes after what seems like your concluding statement. Can you rephrase this part of the abstract to talk about pest suppression as a whole, then maybe a sentence that talks about livestock pests and plant pests? The end of the abstract is disorganized in its current form.

⇨ To avoid confusion in the end of the Abstract, I moved the sentence about general pest suppression before to discuss about the cattle fly pests. As such, the discourse goes from general (all pest species) to precise (only those related to cattle).

Introduction

Line 53: This is getting slightly outdated – use Simmons and Cirranello 2023 (batnames.org). It’s up to almost 1500 species now.

⇨ Thank you for pointing out this outdated information. I did not know about the batnames.org data compilation and would surely use it in the future!

Lines 53-54: Poor sentence structure. Removing the part about European bats and placing it in its own sentence would be better.

⇨ I kept the section about European bats in the same sentence but reformulated it so that the connection is smoother.

Line 55: remove "hence"

⇨ I removed the whole sentence to refocus the topic of this paragraph on bats instead of insects (as intelligently suggested below).

Lines 54-59: It seems that this information here is needed to demonstrate that we need bat diet data to help with bat conservation. I think you should trim this down to one sentence or two at most, because your paragraph oscillates between talking about bats, talking about insects, and then talking about bats again when it seems that the goal of this paragraph is to talk about bats, their diet, and how diet can inform conservation. Therefore, trim some of this information about insects down so that the paragraph mainly focuses on bats.

⇨ Thank you for this suggestion. I streamlined the whole paragraph to make it more impactful.

Line 66-78: A lot of your sentences start with connectors. These are really useful in moderation but when used too frequently they seem unnecessary. Try reading through and see where you can remove some of these connectors to help with sentence flow.

⇨ That is true that I overdo it with connectors. I was not aware of that so thank you for pointing out this issue in my writing. I went through the whole manuscript and removed unnecessary ones.

Line 75: "would be"? Would be consumed if what happened?

⇨ I rephrased this sentence at the past tense.

Line 77: remove "as such"

⇨ I removed it.

Line 77-78: I like this line of thinking!

⇨ Thank you!

Line 83: reword "It was already shown…" to "Previous work shows..."

⇨ I modified it into "Previous studies have shown…".

Lines 83-85: These two sentences can be combined to add some varying sentence structure and remove extra words.

⇨ I merged these two sentences.

Line 86: "In regard to" instead of "As regards…"

⇨ I replaced it as advised.

Line 88: reword to "Most recently, a field experimental study…"

⇨ I rephrased it as such: "In addition, a recent experimental field study…", because I did not want the focus to be on how recent it was.

Line 90: "are threatened by climate change"

⇨ I rephrased this sentence at the present tense.

Line 91: "menace" is fairly emotive. You could try something like "Nearly 60% of European forests are threatened by climate change, with insect outbreaks accounting for 26% of potential damage".

- ⇨ I replaced "menace" by "damage" as advised.

Line 94: "Albeit" is used incorrectly here. References 34 and 35 are not studies on bat regulatory action.

- ⇨ Indeed, references 34 and 35 (now 26 and 27) do not relate to bat regulatory action (first section of the sentence) but to the detrimental effects of both *S. calcitrans* and *M. domestica* on livestock, which constitutes the second section of the sentence. Yet this was confusing and I thus included a reference related to bat regulatory action just after having mentioned it. I also replaced "albeit" by "despite".

Line 97-98: Is "livestock actual welfare regulations" a specific term? If not, remove "actual".

- ⇨ I removed this word. That was a mistake made from a wrong French-English translation. Thank you for having noticing it.

Line 102: both what? You've provided three potential options here; both refers to just two.

- ⇨ "Both" referred to (1) roosts (being either (a) transitory roosts or (b) breeding roosts) and (2) foraging sites. I however acknowledge that it might be unclear (while also unnecessarily detailed) and I therefore reformulated the sentence.

Line 108: reword to "…arises from hunting flexibility and its association with trophic niche breadth.". "the latter" doesn't quite work in this sentence currently and comes off as unnecessary words.

- ⇨ Thank you for this wise rephrasing. I however removed this sentence from the text.

Line 110: "thus" is unnecessary here.

- ⇨ I removed it.

Line 111: "hence" is unnecessary here. Rewording to "Bat species that display various hunting strategies can feed on more diverse prey and use a wide range of foraging grounds, ultimately decreasing their vulnerability to environmental heterogeneity." Would help reduce excessive wordage and with directing the message of your sentence. Also, by heterogeneity, do you mean environmental changes? Many wildlife species require habitat heterogeneity, so this may not be the best word choice for this sentence.

- ⇨ I rewrote the sentence with that relevant suggestion in mind. I also replaced "heterogeneity" by "change".

Line 112: Shouldn't you introduce the different types of hunting styles before stating that bats will use various hunting strategies which reduces their vulnerability to environmental heterogeneity?

- ⇨ Exactly!

Line 116: favored by who? Bats? Or do you mean conserved?

- ⇨ Favored was indeed not the most relevant word choice and I thereby replaced it by "maintained or restored".

Line 117: "also" is not necessary in this sentence.

- ⇨ I deleted it.

Line 118-119: What does the first part of this sentence mean? A close match in space and time? What does this have to do with whether their pests are considered prey?

- ⇨ This consideration was out-of-scope and I therefore reformulated it to focus of the need to study the breeding season variations of the diet rather than the spatio-temporal ones. The purpose of this section: "whether these prey are

considered as pests or not" initially" was in fact to encompass all arthropod prey but it is more confusing than anything else and I therefore removed it.

Line 127: "increased invasion risk of pests"… This part of the sentence does not make sense with the beginning of the sentence… what are you trying to say exactly?

- ⇨ I cut-out a large part of this paragraph as it was just disorienting the focus of the main story.

Line 143: This study does not employ a wide spatio-temporal scale. The spatial replication is restricted to part of Belgium and the temporal scale is across three time points in the breeding season. The language about this scale needs to be adjusted throughout the manuscript.

- ⇨ As stated above, I refocused the goal to study the breeding season variations of the diet in its northern range rather than its spatio-temporal variations.

Line 155: I think you can say that you will discuss the potential hunting strategies and foraging habitats used by *M. emarginatus*. You don't explicitly determine these behaviors given the current data, but you can have a section that discusses the potential strategies used by bats.

- ⇨ I reformulated this sentence to avoid overplaying the implications of this work.

Line 165-169: This sounds like content for the Discussion.

- ⇨ That's right. I incorporated this section to the Discussion.

<u>Material & Methods</u>

Line 189: Why are you abbreviating to ME now instead of earlier in the manuscript?

- ⇨ Very good question. I modified it so that the first abbreviation occurs in the Introduction following the description of the research goals. However, I kept the unabbreviated species name at the beginning of the Discussion and Conclusion as a reminder.

Line 192: Need to explicitly define the temporal limits of each session. Also need to provide biological reasoning for why you delineated the breeding season into these sessions. You state in the discussion that these loosely align with breeding, lactation, and parturition periods but need to state here.

- ⇨ I detailed thoroughly the temporal delimitations of each session and to which breeding stage they correspond.

Line 196: why does it matter that *R. ferrumequinum* co-roosts with this species?

- ⇨ It does not matter but I found this ecological consideration interesting to mention. However, it is not crucial information and I thus moved it to the S1 File, which contains the supplementary Methods.

Line 197: Reformat references.

- ⇨ Thank you for pinpointing this.

Line 204: Remove "being" – not needed in the sentence.

- ⇨ I removed it.

Line 205: Product information for the DNA decontaminant.

- ⇨ I added this specification.

Line 207: So because this is under-roost sampling that cannot be traced back to individuals, you can't really make claims like Line 38 about 55% of females foraging in cowsheds. You have no way of knowing that these fecal samples didn't all come from a few bats as you do not discuss testing the bat DNA of the feces.

- ⇨ I fully agree with this drawback of my work. That was a wrong assumption to consider that 1 pellet = 1 individual. As such, I reformulated "bat individuals" to "individual bat pellets" or simply retained the term "samples" throughout the whole manuscript.

Line 239: What do you mean "to trace back samples' origin" – like to see what bat it came from? What species? Further explanation is needed.

- ⇨ In Illumina protocols, PCR products amplified with a specific primer pair (e.g. either Galan or Zeale) are pooled together (i.e. multiplexed) before sequencing. As consequence, the use of specific combination of tags for each sample is necessary "to traced samples' ID", which means to reassign DNA sequences to the sample they originate from in multiplexing protocols. I described this in details in the S1 File, which contains additional Methods.

Line 258: What are ASVs?

- ⇨ Amplicon Sequence Variant; I explained this abbreviation.

Line 282: You need to state here or somewhere in the manuscript the number of samples that were analyzed from each site and from each session.

- ⇨ I added a graphic displaying the number of samples analyzed per sampling session and colony (S2 Fig).

Line 290: You can't say this based on your proposed methods, which state that you did not do any DNA testing confirming that fecal samples came from individual bats.

- ⇨ As recommended and to be more honest in relation to the available data, I removed any inferences about the number of individual bats.

Line 313: re-arrange to "We used the number of taxa identified per order in each sample as the response variable."

- ⇨ I instead rewrote the sentence like this: "The response variable was the number of taxa identified per order in each sample." This allows me to keep "the response variable" as the subject of the sentence and detailing it afterwards.

Line 334: Should try to stay consistent with terminology – spiders vs. Aranae. Also, what is meant by closed-landscapes? Like closed-canopy?

- ⇨ Thank you for this comment. Overall, I replaced "spider" by "Araneae" in the whole manuscript except when mentioning the spider guilds as this terminology has been established by Uetz et al. in 1999 (see reference [89] in the manuscript).

Line 356: What are the criteria for denoting a species as a minor or major pest? None listed.

- ⇨ I added this information in the S1 File, which contains the supplementary Methods.

Line 366: This sentence needs to be incorporated somewhere as it is a single sentence that can't stand alone as a paragraph.

- ⇨ I merged it to the previous paragraph.

<u>Results</u>

Line 402: Which level of taxonomy is this sentence referring to? You've talked about several different taxonomic levels so far, so it is unclear which is being discussed here. If stated previously, I suggest refreshing the reader's mind and adding a statement here about which taxonomic levels were analysed in this section.

⇨ I did not add a reminder within the results but I stated explicitly in the Methods what is encompassed by the term "taxa": "Throughout this manuscript, the "taxa" term has been used to encompass several taxonomic levels, but always with the most precise identification achievable." (see lines 298-300).

Line 410: I feel like this makes sense as the variation in temporal data collection points is very limited. Could be a good discussion point.

⇨ Thank you for raising this point. I mentioned it into account in the Discussion (see lines 645-646). The fact that none of the localities' curves reached a plateau could indeed be due to the sparse temporal sampling (3 months) as compared to the entire period during which bats were awake during the year (i.e. overall spanning seven months, generally from April to October in the considered species in the study area). However, it is quite common in metabarcoding studies to not reach the plateau and I also included some references supporting this general issue (see lines 646-647).

Line 415: reword to "In regard to sample coverage curves,"

⇨ I removed the analysis of sample coverage as it was both ambiguous and unnecessary.

Line 417: "overall good capturing" is not research-grade English and I suggest rewording.

⇨ As stated just above, I removed this section but thank you for the advice.

Line 420: These sentiments provide evidence that your data does not provide much in the way of spatial variation.

⇨ That is true and that is partly why I decided – according to your suggestion – to cut out the spatial variation analysis from the main text. I kept it as supplementary data though (see the S4 File) but only to verify the absence of large spatial variations in the diet between the 5 maternity colonies in the northern edge of the species range.

Line 499: "In regard to variation in diet composition". "As regards" only works in certain settings, and I don't think it fits here. Better yet – remove "In regard" and try "Our NMDS plot showed that locality was not a major driver of variation in diet composition (Fig 6)." There are a lot of areas throughout the document that can remove unnecessary words to help with the directness of phrasing and increase reader comprehension.

⇨ Thank you again for your help on improving my English and the conciseness of this work. However, in this precise case, I think that the longer version still holds the best potential for reader comprehension as it reminds what we were looking for before to describe the outputs of the analysis. I therefore went for the first option by starting the sentence with the "in regard to" reformulation you suggested.

Lines 516-518: What does this statistic tell the reader? Just that these two things are correlated, which does not provide sufficient evidence to show that bats are foraging for these spiders in forests.

⇨ To make this work clearer but also more compelling, I removed this analysis, which aimed to evaluate whether eaten spiders were associated to closed-landscapes (i.e. natural cluttered environments such as forests). Far from being uninteresting *per se*, this section did not exactly fit into the goals and I therefore preferred deleting it.

Line 535: You cannot say 163 individual bats because from the methods, it does not sound like samples were collected from individuals – they were collected from under roosts. If you provide any genetic analyses showing that fecal samples are from individual bats, then that needs to be presented. But in its current form, you cannot state anything about samples from individuals.

⇨ I did not perform any individualizing analyses. Therefore, I removed any inferences about the number of individual bats to be more honest in relation to the available data.

Line 537: Same thing here about discussing individuals.

⇨ As recommended and to be more honest in relation to the available data, I removed any inferences about the number of individual bats.

Line 558: Same thing here about discussing individuals.

- ⇨ As recommended and to be more honest in relation to the available data, I removed any inferences about the number of individual bats.

Line 582: 55.38%

- ⇨ That is correct, thank you.

Line 584-585: What is this statistic supposed to demonstrate exactly? Similar to my comment above, it is just looking at correlation without any additional data to make it biologically meaningful in this situation. Would cow presence be more useful in this situation? As you could show that fly presence is correlated with cow presence at least? Regardless, this is still not evidence for the foraging in cowsheds claim.

- ⇨ I acknowledge that it is "just" correlative data and is thus not proving anything. Anyhow, science is also about being speculative and posing some intuitive hypotheses. In addition, I here attested of cow DNA presence within the same samples in which we detected DNA of cattle-related flies (i.e. *Musca domestica* and *Stomoxys calcitrans*). For this analysis, I <u>only</u> used this reduced set of samples in which congruent identification (both cow and flies DNA) occurred. It might however not have been clear because I did not remind this crucial information in the lines 584-585. I now filled this gap by adding this "that had been attested to feed on cows" to the sentence.
- ⇨ Furthermore, we selected samples containing cow DNA by applying strict sequencing filters. Thus, *M. emarginatus* <u>must</u> have caught these flies around cattle or at some distance to it since "flight range for flies (house flies) released from stable farm was 5 km" (Nazni et al. 2005). Anyhow, *M. emarginatus* bats seem to extensively rely on these flies and I therefore have serious doubt that these bats preyed upon cattle flies by just hazardous encounters in the landscape. Instead, I suggest (I toned down by claims by being more speculative than assertive) that capturing these flies would occur in and around cowsheds, in which the concentration of cattle flies is massive (see Krull et al. 1991; reference [59] in the manuscript).
- ⇨ Keeping this in mind when interpreting the Figure 8, I found it surprising that cattle flies did not represent a greater proportion of the diet (wPO metric) in the samples collected from cattle-rich as compared to cattle-poor areas. I therefore proposed that the absence of correlation indicates that *M. emarginatus* would rely on cattle flies no matter the opportunity for foraging around cattle. In all cases, I tempered my claims by suggesting that catching these flies could occur in and around cowsheds, rather than limiting my conclusion to cowsheds only. Thank you for advising me to tone down my interpretations as it is more accurate given the data I presented.

<u>Discussion</u>

Line 607: The temporal and spatial coverage of your samples are not extensive, I suggest rephrasing.

- ⇨ I rephrased according to the reformulated objectives.

Line 614: No supporting evidence for this claim.

- ⇨ I tempered my claims by including "We suggest that"

Line 616: No supporting evidence for this claim.

- ⇨ I tempered my claims by including "We speculate that"

Line 617: I suggest a more appropriate term for research instead of "great". Maybe "thorough"? Or even "excellent"? "High-quality"?

- ⇨ I replaced "great" by "extensive".

Line 649: I think this is a great way to frame your findings! You looked at the regional diet of ME, which is important because this is the northernmost point of their range. That claim is actually supported by the data you present here and could make for a compelling story.

- ⇨ Thank you very much for this insightful suggestion; this is indeed what I did.

Line 651: Each sample, not each bat because there is no statement or data showing that each sample came from an individual bat – only that you tried to get them from individuals based on where they were roosting. That is not the same as definitively collecting samples from individuals.

⇨ As recommended and to be more honest in relation to the available data, I removed any inferences about the number of individual bats.

Line 701: individual samples, not bats.

⇨ As recommended and to be more honest in relation to the available data, I removed any inferences about the number of individual bats.

Line 712-714: This is an important point as it shows that your sites do not really represent spatial variation relative to the range of this bat species.

⇨ That is entirely correct and, as recommended, I reformulated my objectives to focus on the breeding season variations in diet at the northern limit of the range of *M. emarginatus*, instead of testing for spatial differences across the colonies. Rather than expecting spatial variation in diet, I now expect a similar diet across the (quite small) study area.

Line 721-723: Why is this single sentence a separate paragraph? There are several cases of this throughout the manuscript that need to be addressed as a single sentence typically cannot be a paragraph on its own.

⇨ I removed this sentence from the manuscript but I went through the whole manuscript to fix this problem of single sentence paragraphs.

Line 755: Provide a percentage for how often cow DNA was detected in samples with flies.

⇨ I added this percentage for the months of July and August together as they corresponded to the detection peaks of cattle flies in the guano samples (see lines 577 and 753).

Line 756: Cannot say use of cowsheds for foraging as you have no evidence. That may be where the cows the flies prey upon are, but flies can disperse over several kilometers, meaning they are not limited to cowsheds.

⇨ I moderated by claim by inserting "cowsheds and their vicinity" because even though (house) flies are effectively able to disperse from their origin up to 7 km (Nazni et al. 2005), I doubt that their concentration at such a distance would allow ME to rely on them at such a great extent. Indeed, Diptera accounted for 40-50% of the wPO metric in July and August and cattle-flies' DNA was retrieved in 75.97% of the samples from July and August, suggesting that these bats effectively and extensively rely upon cattle.

Lines 818-821: Why is this a separate paragraph? It's not developed enough to stand on its own in its current form.

⇨ In fact, the section "E. *M. emarginatus* is an agile gleaner forest forager" develops into 3 topics: agility, gleaner, forest.

⇨ The first paragraph develops the "gleaner" hunting strategy, the second one expands on the "forest" habitat type and the third one (the one mentioned here) informs about the "agile" flight of *M. emarginatus*. By merging the third paragraph to the previous one, I am afraid it would disorient the reader and I thus chose to keep it as a single paragraph to avoid confusion.

Lines 822-823: There is no evidence confirming foraging in these areas. You can say that they may forage in these areas based on captured prey, but as I've mentioned previously these fly species are not restricted to only occurring in cowsheds and there is huge variety in where spiders and lepidopterans may live. Further, you do not provide much context for the surrounding landscapes, so it is hard to infer if these bats are selecting these specific foraging regions you mention or if they are just foraging based on what's available.

⇨ I removed this sentence as it was indeed an overstatement. In addition, I created a map (Fig 1) depicting nine landcover classes and their relative proportion around each roost in a related graph (Fig 2) to help understanding the landscape in which this study occurred.

Line 823: As you state here, you inferred these claims. That is fine, but you need to be up front that these are inference-based claims, not data-driven claims. I do think there is room with your data to provide such suggestions that bats may forage in cowsheds and these other areas, but you currently do not have the data to be so specific with your claims.

- ⇨ I moderated this assessment by mentioning this: "Although ME foraging habitat and behavior were inferred from the ecology of their prey, we are confident that this species likely exploited these habitats for feeding.".

Line 838: What do these numbers next to minor and major mean? Are those the number of taxa in each category?

- ⇨ Yes, they correspond to the number of taxa. I made it more obvious.

Reviewer #3:

Review of the manuscript PONE-D-23-33429 "All-you-can-eat buffet: A spider-specialized bat species (*Myotis emarginatus*) turns into a pest flies' eater in cowsheds. In this investigation, the authors investigated diet composition, its spatio-temporal variations, foraging behaviour, and pest hunting of *Myotis emarginatus* from six location in Wallonia, Belgium. Overall, I found the manuscript too lengthy with very confused objectives and methods. I think the most interesting result found by the authors is the fact that bats complement or shift their diet with Diptera in the most energetic demanding phase of reproduction. I recommend the authors to reduce the length of the manuscript by focusing in this main result. I have made some recommendations below.

- ⇨ Thank you very much for this thoughtful review of my manuscript. I streamlined the objectives and removed the focus on the spatial variation to focus on the breeding season variations in diet of *M. emarginatus* at the northern edge of its range. I also investigated the pest consumption of this bat species (this goal has not changed). Finally, I inferred the foraging habitats and hunting strategies of this bat species from the prey ecological traits. This final goal has not changed neither but I tempered my claims to avoid overplaying my findings. As such, I included many terms like "presumably", "likely", "we suggest/speculate that", … In general, I reminded the aims of the study all along the manuscript, whether for describing the purpose of the Methods of for replacing the Results in the context of research. This refining gives a clearer direction to the paper. I finally did an intense work of cutting-out unnecessary paragraphs/words and fillers to move towards clarity and conciseness.

<u>Introduction</u>

I find the introduction quite extensive and jumpy. The authors use a broad number of terms that are not properly defined, which makes the reading confusing. In the text, global warming is largely mentioned. Nevertheless, the reduction in entomofauna due global warming goes out of the scope of the investigation, as the authors did not take any long-term measurement to establish a metric of this phenomena. I suggest reduce the length of the introduction by eliminating all the information regarding global warming and entomofauna reduction.

- ⇨ As advised, I downsized the length of the Introduction. However, I think that mentioning global warming and its implications on entomofauna still remains interesting. Of course, I did not study changes in arthropod populations in the face of global warming. Yet these allusions to environmental changes replace this study into a broader context before to scale down and to describe the current knowledge gaps and how this work could help filling them.
    - o Starting from the mention of global warming and its consequences on arthropods, I first postulated that studying the diet of insectivorous bats was necessary to evaluate whether they are threatened by the decline in insect density/diversity.
    - o Second, I described that human footprint favors some insect species – pest species – despite this drop in insects. I built upon this problem and advanced that bats could be used as pest control but could also be very helpful to uncover rare and new arthropod species (i.e. natural samplers).
    - o I further described the type of habitats for which evidence of pest consumption was currently lacking: forests and cattle-farming landscapes. Following this, I posited that hunting flexibility would improve bats resistance to environmental changes.
    - o As concerns the importance to study the bat diet along the breeding season, it is quite clear that changes in insect phenology owing to climate change could have large impacts on the availability of insect items and thereby on diet composition.
- ⇨ Overall, even though we did not quantify human-associated massive decline in arthropods, I think that this global issue offers a solid basis on which the objectives of this work will build upon.

<u>Specific observations</u>

L 43-44. What do you mean the cowsheds act as ecological traps? I think this does not make much sense as is presented in this part of the manuscript.

- ⇨ I removed this sentence from the Abstract.

L 59. "and food provisioning"

- ⇨ I removed this sentence from the Introduction.

L 76-78. I do like this idea showing bats as samplers of biodiversity. Nevertheless, I would discard this idea because in such case the sampling would be biased by the preferences of prey by the different species composing the order.

⇨ I acknowledge that there is an inherent bias related to bats dietary spectrum and preferences. However, bearing that drawback in mind, I still believe that insectivorous bats may prove useful to uncover the presence of insect species. Of course, the more diverse the studied bats will be in terms of hunting strategy, size and foraging habitats, the better the whole landscape will be naturally sampled. Yet we attest here the interest of investigating bat diets with metabarcoding approach as we discovered a whole range of potential new arthropod species at the Walloon scale (~70 species; see the S3 File).

L 84. Is this species a pest in the pine forest?

⇨ Yes, indeed. See the associated reference in the manuscript ([22]) and further information in this report written by the EPPO, i.e. European and Mediterranean Plant Protection Organization (https://gd.eppo.int/taxon/THAUPI/download/datasheet_pdf), which describes this:

"*T. pityocampa* is one of the most important forest pests throughout the temperate regions of the Mediterranean basin, infesting mainly pine but also cedar trees (the latter in North Africa). Defoliation caused by *T. pityocampa* significantly impairs the vitality of host trees, affecting both their growth (Cadahía and Insua, 1970; Carus, 2004; Jacquet et al., 2013) and their susceptibility to other secondary, bark and wood boring insects (Masutti & Battisti, 1990; Zamoun, 2002).

L 96. By disturbing feeding of "livestock" and their ability…

⇨ I rephrased this section of the paragraph and recalled the term "livestock" as suggested.

L 97. I cannot understand how *S. calcitrans* could reduce meat production?

⇨ I added more details to this section to improve comprehension (see lines 88-91).

L 103 What topic?

⇨ The topic refers to "bat feeding on pest flies in farmland areas". I reformulated this sentence to make it clearer (see lines 96-98).

L 105-106. If bats do not restrict their foraging activity to a single habitat, how cattle-related "habitats" can be so hazardous for them (L 97-100)

⇨ In fact, cattle farms are generally attractive for bats at first sight, because they host plenty of food (see Krull et al. 1991; reference [59] in the manuscript: "Numerous muscid and drosophilid flies covered the ceiling with densities of 10-40 and 100-400 flies/m^2, respectively"). Indeed, insecticide application is almost never sufficient to effectively reduce flies' populations. However, these flies may still contain hazardous chemical compounds that will be ingested by bats when these feed on contaminated flies. The consequences of this ingestion are currently essentially unknown. However, there is a risk that cowsheds could act as ecological traps for bats if these become secondary poisoned.

To support my claim, here are two extracts of reviews on the risk associated to *S. calcitrans*.

⇨ From Baldacchino et al. 2013 (see reference [26] in the manuscript): "Insecticides applied as residual sprays, such as permethrin, have been applied to the sides of buildings where adult stable flies congregate. However, the persistence of residuals is not predictable over time. Sublethal exposure levels may be found less than one month after application [60] and some stable fly populations have already shown a high degree of resistance [102]."

⇨ From Patra et al. 2018 (see reference [27] in the manuscript): "However, many of the insecticide are short lived and treatment of large number of animals using pesticides is impractical unless stable fly population is extremely high. […] Unfortunately, overtime stable fly becomes resistant to the pesticides used (Salem et al., 2012)."

L108 What "hunting flexibility" mean?

- ⇨ I removed this sentence but defined appropriately hunting flexibility and its implications in the following sentence (see lines 104-106).

L How are "hunting strategies" and "hunting stiles" (L112) alike or not?

- ⇨ Yes, they are. To be more consistent, I used the "hunting strategies" formulation throughout the manuscript.

L 111-112 "hence decreasing their vulnerability to environmental heterogeneity". What do you mean with this? Please clarify

- ⇨ I reformulated this sentence to improve comprehension (see lines 104-106).

L 111, 114. What do you mean by foraging grounds?

- ⇨ I mean foraging habitats. I changed it in its first instance.

L 127-129. Yet, this was not the main goal to study in your investigation.

- ⇨ That is true and that is the reason why I downsized this whole paragraph and refocused its topic on the importance of studying the breeding season variations of insectivorous bat diets (see lines 111-117).

L 165-169. This information should be put in the discussion section of the manuscript, not here.

- ⇨ Indeed, I integrated into the Discussion.

<u>Methods</u>

The methods section is difficult to follow as it has many undefined variables that the authors used for the different analyses. The methods are also jumpy and poorly understandable. Most of the analysis do not have any support to fulfill the objectives pursued by the investigation. I suggest to present the main objectives and the analysis related to these objectives in one paragraph and then assess them in detail in the order that were presented in the following paragraphs.

- ⇨ As stated above, I followed your wise suggestion to remind the objectives first and develop the analyses associated to it afterwards. I also reworked the S1 File which contains the supplementary Methods in a way that directly linked each section to the related paragraph in the Methods (by using subtitles and clear opening sentences).

Some information about the different breeding colonies is missing. Are those sites surrounded by vegetation or rather immerse in cities? If that's so, which kind of vegetation was? How far are they separated each from the other? I think this information is relevant because is related to prey availability which is related to the main objectives of the investigation.

- ⇨ Thank you for pinpointing this drawback in our study. To fill this gap, I created a map (Fig 1) depicting nine landcover classes and their relative proportion around each roost in a related graphic (Fig 2). I also described the main tree species composing broadleaved forests, the dominating types of crops and the function of managed grasslands. Finally, the distance between roosts is noted in the S1 File, which describes additional Methods. This file also gives further details on the study area such as the minimum distance between roosts, mean precipitations, altitude, biogeographic regions, …

I would reduce the number of acronyms used because is difficult to follow them along the manuscript.

- ⇨ I removed the abbreviations related to the session (S1, S2 and S3) and instead stick to the corresponding month, i.e. June, July and August.

Please add package citations among the methods.

- ⇨ Citations are now included following every mention of the packages. That is also true for the S1 File.

<u>Specific observations</u>

L 188-189. *Myotis emarginatus* must be abbreviated as *M. emarginatus* not ME.

⇨ Since *M. emarginatus* remains a lengthy term, I would rather use ME to improve the fluency of the manuscript.

L 192-193. It would be easier naming the sessions as the months (June, July, August) instead of S1, S2 and S3

⇨ That is true! I modified it accordingly.

L 285-295. Were all those metrics calculated for the whole monitoring? Specify

⇨ I added a sentence to explain that these metrics were used on the whole dataset of samples but also on subsets of it depending on the objectives (see lines 314-316). Further in the Methods, I always mentioned which (sub)dataset was used.

L 299-300. What was the purpose of doing these calculations for each order?

⇨ The wPO metric, i.e. the weighted percentage of occurrence correspond to:
Prey item occurrence/total number of occurrences of **all** prey*100 (within each sample)
Thus, this metric applied to the Lepidoptera order for instance, would be calculated a such:
Prey item occurrence of a **Lepidoptera** taxa/total number of occurrences of **Lepidoptera** prey*100 (within each sample containing at least one taxa of Lepidoptera)

⇨ While the numerator remains the prey taxa occurrence (= 1), the denominator widely changes depending on the considered dataset.

L 307. What was the temporality you are talking about? wasn't the study done only during the breeding season (L188)

⇨ Yes, indeed. As consequence, I replaced "temporality" by "session (i.e. sampling month)" to improve the clarity of our aims. I also reminded the objective just before to detail the analysis that would help answer our question.

L 309. "localities/sessions" is the session each one of the visits you performed? the diagonal between those variables indicates that they were nested in the analyses?

⇨ This analysis (sample coverage) was ambiguous and unnecessary and I therefore removed it.

L 310. What was the sample coverage?

⇨ This analysis was ambiguous and unnecessary and I therefore removed it.

L 313. When you refer to taxa you mean species fully identified?

⇨ I acknowledge that it lacks clarity. Therefore, I explicitly described in the Methods what the taxa consists in: "Throughout this manuscript, the "taxa" term has been used to encompass several taxonomic levels, but always with the most precise identification achievable." (see lines 298-300).

L 314-315. "When locality/session was introduced as a fixed factor, we considered session/locality as a random factor" to what end? Explain

⇨ The **Fixed** Effects are the predictors that have a fixed and constant effect on the response variable.
The **Random** Effects are additional sources of variability that are not fixed but are specific to certain groups or clusters in the data.

⇨ Here, I ran 2 types of models (but moved the results of those where "locality = fixed variable to the S4 File) depending on whether I considered the session/locality as the fixed or random effect.
  - Thus, when assessing the **impact of the 3 sessions** on the mean richness of prey at the order level, I also wanted to capture the variability between samples that was not explained by the session factor but which was attributed to the locality.
  - Conversely, when assessing the **impact of the 5 localities** on the mean richness of prey at the order level, I also wanted to capture the variability between samples that was not explained by the locality factor but which was attributed to the session.

L 319. "Then, we tested the impact of spatial and temporal variation" Which quantifiable variables were those?

- ⇨ When investigating the temporal variation, it concerned the session (i.e. the months of June, July and August) variable.
  When investigating the spatial variation, it concerned the locality (i.e. the 5 breeding colonies) variable (see the S4 File for the results of this analysis).
- ⇨ To improve clarity, I replaced the terms temporal/spatial by session/locality.

L 339-342. "To get insights into spiders' ecology" what this does mean? Also, what was the end of characterizing functional groups of spiders related to the main objective of the investigation?

- ⇨ I again started this section (Ecological traits of arthropod prey species) by reformulating the related goals to improve the reader's comprehension (see lines 359-362).

L 343. Does this mean you performed a spiders monitoring? How was this conducted? This is not presented in any part of the manuscript.

- ⇨ I did not perform any spiders monitoring. Instead, I conducted and extensive bibliographic research to gather information about spiders' ecology (i.e. hunting strategy and type of web), which gave me information about the habitat presumably occupied by each taxon of spider. As gleaning bats most likely catch their prey in the habitat occupied by the prey, it allowed me to infer which habitats could be used by *M. emarginatus* for foraging, based on the spider prey taxa it consumed. This is inference data and I acknowledge that it has drawbacks and biases but it also holds strong potential for unveiling landscape occupation by cryptic and nocturnal mammals such as temperate insectivorous bats.

L 348-351. I do not understand this at all. What was the end of doing so? Which were the response and explanatory variables? What was the nature (i.e. numerical or categorical) of the data? If some variable was categorical, how many levels does it had?

- ⇨ The idea was initially to assess whether Lepidoptera species in the larval stage could be found in similar proportion across all colonies. Yet I preferred removing this analysis as it was not answering any hypothesis, hence disrupting the flow of the Methods section.

L 353. Eliminate repeated word.

- ⇨ These are not repetitions but two distinct words (through and thorough) but I replaced "thorough" by "an in-depth" to facilitate the reader's task.

L 366. Statistical differences are stablished when alpha and not p value are < 0.05.

- ⇨ I modified it accordingly.

<u>Discussion</u>

L 612-613. It should be parturition and lactation period.

- ⇨ Thank you for this comment. I modified it throughout the whole paper.

L 614-616. I wonder why the authors are sure to assert that the house flies are hyper abundant in the cattle sheds since they did not take any measurement during the performance of the study.

- ⇨ I certainly acknowledge that we did not measure fly abundance around cattle. However, several points allow me to suggest that cattle flies prevail in the vicinity of cowsheds which become thus attractive for ME bats.
  - First, I here attested of cow DNA presence within the samples containing DNA of cattle-related flies (i.e. *Musca domestica* and *Stomoxys calcitrans*). For some analyses (Kruskall-Wallis tests, Pearson correlation; see lines 383-386 and 388-391), I used this reduced set of samples in which congruent identification (of both cow and flies DNA) occurred. Furthermore, we selected samples containing DNA from cattle applying strict sequencing filters. Thus, *M. emarginatus* must have caught these flies around cattle.
  - Second, house flies hyperabundance has been mentioned in the paper by Krull et al. 1991 (see reference [59] in the manuscript).
  - Third, *S. calcitrans* is also commonly called "stable fly" or "barn fly" and I considered this appellation to be self-explaining.

- ⇨ In spite of this, asserting that ME bats hunt in cowsheds is an overstatement. Thank you for raising this issue. Therefore, I tempered my claims by mentioning throughout the manuscript that bats may have foraged in and around cowsheds. Also, I used words like "suggest" or "speculate" to avoid overextending on my results.

L 617-662. I would reduce this section of the manuscript.

- ⇨ I removed a whole paragraph of this section.

L 618. While I agree that metabarcoding is a good technique to assess diet in bats, I am not sure that insectivorous bats would be excellent natural samplers of biodiversity. As I stated above, prey consumed by bats may depend of different preferences of the species (many of them linked to the animals' physiology). Also, because bats travel a variety of distances and habitats in a single night to forage, we could not be able to associate concrete areas holding such diversity. This may difficult the interpretation of the diversity patterns related to the ecosystems.

- ⇨ I rephrased this first sentence to remove the focus from bats being good natural samplers: "The diet of insectivorous bat species such as ME can be thoroughly described using a dual-primer metabarcoding approach".

L 617. I think that the discussion of the method should be moved to the last

- ⇨ I think both options (either at first or at last) are valuable are have their own drawbacks.
  - By discussing the methods in the first place, it may distract the impatient reader who is waiting for the flesh of the story, that is, the discussion pertaining to the four objectives.
  - By discussing them at last however, the reader would last with the feeling that the main story ended before and that this last section is just a bunch of unnecessary details that were packed there after all the interesting stuffs. In this situation, there is a high risk that the reader simply jumps over this last paragraph, despite its interest. It will also leave the reader with the idea that we missed something, rather than leaving him/her with a feeling of scientific achievement.
- ⇨ Thus, I decided to start with the discussion of the methods. In fact, the comparison of the primers from Galan versus Zeale corresponds to the first results I obtained following the read filtering and data pre-processing. The order of reading thus corresponds to the analysis steps, thereby enhancing reading flow.
- ⇨ I am thus betting that readers will be curious enough to go through this first paragraph to discover the inner story and main conclusions of this work.

L 620-621. Were those studies performed in the same area and temporality? Differences in prey consumed could be resulted just because differences on these characteristics.

- ⇨ I added the study area and the temporality of these research.

L 660-662. See my previous comment on this regard.

- ⇨ I fully agree with this drawback. However, I still believe that bats hold strong potential for sampling the biodiversity but at a more regional scale and I rephrase the sentence to include this notion. For instance, at the Walloon regional scale, we discovered in this work a whole range of potential new arthropods species (~70 species; see the S3 File).

L 724-737. This is the reason because I asked before for the characteristics of the shelters to be described in the methods section. Differences in the areas surrounding the shelters may explain differences in the food items presents in the pellets of individuals.

- ⇨ As mentioned earlier in this file, I removed the analysis concerning the link between catching spiders and the percentage of forest surrounding each roost.

L 885-881. I would reduce this section of the manuscript.

- ⇨ I downsized this section.

L 882-901. I would reduce this section of the manuscript.

- ⇨ This section is now almost half its initial size.

6. PLOS authors have the option to publish the peer review history of their article (what does this mean?). If published, this will include your full peer review and any attached files.

If you choose “no”, your identity will remain anonymous but your review may still be made public.

Do you want your identity to be public for this peer review? For information about this choice, including consent withdrawal, please see our Privacy Policy.

Reviewer #1: No

Reviewer #2: No

Reviewer #3: Yes

[NOTE: If reviewer comments were submitted as an attachment file, they will be attached to this email and accessible via the submission site. Please log into your account, locate the manuscript record, and check for the action link "View Attachments". If this link does not appear, there are no attachment files.]

While revising your submission, please upload your figure files to the Preflight Analysis and Conversion Engine (PACE) digital diagnostic tool, https://pacev2.apexcovantage.com/. PACE helps ensure that figures meet PLOS requirements. To use PACE, you must first register as a user. Registration is free. Then, login and navigate to the UPLOAD tab, where you will find detailed instructions on how to use the tool. If you encounter any issues or have any questions when using PACE, please email PLOS at figures@plos.org. Please note that Supporting Information files do not need this step.

In compliance with data protection regulations, you may request that we remove your personal registration details at any time. (Remove my information/details). Please contact the publication office if you have any questions.
